# A Hybrid Hierarchical Framework for Quantifying Mechanical Markers in Atherosclerotic Disease Progression: A New Approach for Diagnosis and Risk Assessment

**DOI:** 10.1002/advs.77995

**Published:** 2026-09-30

**Authors:** Yidan Zhou, Zhuo Chang, Xianjun Wu, Sijie Wang, Yuzhi Zhang, Hao Zhang, Haoyu Xu, Ruining Peng, Zhe Wang, Huiyun Xu, Yu Liu, Guangkui Xu, Nu Zhang, Hui Yang

**Affiliations:** ^1^ School of Life Sciences and Technology Northwestern Polytechnical University Shaanxi China; ^2^ Treatment and Protection Technology and Equipment Engineering Research Center of the Chinese Ministry of Education for Biological Diagnosis Shaanxi China; ^3^ Research Center of Special Environmental Biomechanics & Medical Engineering Northwestern Polytechnical University Shaanxi China; ^4^ Laboratory For Multiscale Mechanics and Medical Science State Key Laboratory For Strength and Vibration of Mechanical Structures School of Aerospace Engineering Xi'an Jiaotong University Shaanxi China; ^5^ School of Physical Science and Technology Northwestern Polytechnical University Shaanxi China; ^6^ Department of Cardiology Nanjing University Medical School Affiliated Nanjing Drum Tower Hospital Jiangsu China

**Keywords:** atherosclerosis, multiscale mechanics, viscoelastic properties

## Abstract

Atherosclerosis is the primary pathological basis for cardiovascular diseases. Despite advances in diagnosis and treatment, current methods primarily focus on late‐stage intervention, missing critical opportunities for early detection. Recently, the dynamic mechanical properties of arterial tissue have been recognized as a novel approach for assessing vascular status, and offer an opportunity to employ the mechanical properties of atherosclerotic lesions for evaluating disease status and monitoring disease progression. Here, we employed a Hybrid Hierarchical theory–Microrheology (HHM) framework to quantify multiscale mechanical markers during lesion progression. The results revealed a two‐stage power‐law rheology, with short‐ and long‐timescale exponents (*α_short_*, *α_long_*) as key mechanical markers of lesion composition and mechanical gradients. We further applied a self‐similar hierarchical framework to capture plaque heterogeneity across cytoplasmic, cellular, and tissue scales, yielding additional mechanical markers (e.g., *E_1_*, *η*, *E_2_*, *E_3_*) from subcellular to tissue scales. Based on these markers, we built a multiparametric diagnostic model that outperforms traditional elastic modulus criterion. This model captures stage‐ and region‐specific trajectories, coupling mechanical signatures with histology for improved staging and risk assessment, establishing an operational framework for enhanced prediction and diagnosis of atherosclerosis.

## Graphical abstract

The mechanobiological basis of atherosclerotic progression and its contribution to spatial and temporal heterogeneity remain incompletely understood. Spatially resolved AFM measurements revealed that plaque formation and distinct regions (cap fibrosis, intimal fibrosis, and lipid‐rich core) exhibit heterogeneous elastic modulus and viscoelastic properties. A multiscale framework integrating cellular composition, extracellular matrix (ECM) remodeling, and mechanical evolution was established, highlighting a progressive transition from a fluid‐like to a solid‐like mechanical state during atherosclerotic development. This transition is accompanied by collagen accumulation, inflammatory cell infiltration, and lipid deposition, which collectively reshape the local mechanical microenvironment. These findings demonstrate that viscoelastic remodeling is a defining feature of plaque progression. Targeting the mechanical microenvironment represents a promising strategy for enhancing the assessment and treatment of atherosclerotic disease.

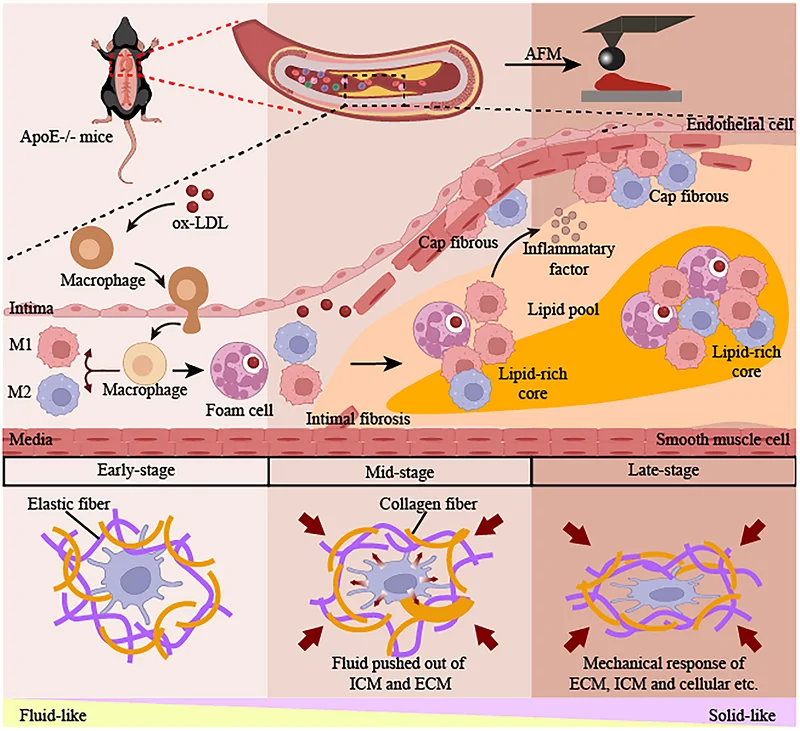

## 1 Introduction

Atherosclerosis is a chronic inflammatory disorder and the primary pathological basis for various fatal cardiovascular diseases (CVDs) [1, 2]. It is characterized by the lipid deposition, plaque formation within the arterial walls and pathological remodeling of vascular tissue [1]. With the development of atherosclerosis, progressive arterial stiffening perturbs the mechanical properties of vascular tissue, which in turn recruits immune cells and facilitates inflammatory infiltration [2, 3]. These abnormal changes in the vascular mechanical microenvironments are closely associated with the risk of rupture, which leads to the progression of thrombus‐mediated acute coronary syndromes [4, 5]. Hence, the mechanical properties of vascular tissues during atherosclerosis are believed to serve as robust markers for accurate disease staging [6, 7, 8, 9, 10].

To date, current clinical management still emphasizes late‐stage intervention (e.g., revascularization and antithrombotic therapy) rather than early, mechanics‐informed detection [4, 5, 11]. Conventional angiography and circulating biomarkers often identify advanced disease; their limitations for early risk stratification often result in delayed CVD diagnoses and increased heart failure risk [6, 7, 12, 13]. Recent advances in detecting static mechanical properties, typically the elastic stiffness, have significantly enhanced our understanding of the heterogeneity within atherosclerotic plaques at different disease stages [8, 9]. However, this single‐parameter approach underrepresents time‐dependent responses, neglects spatial heterogeneity within plaques, and limits the translation into robust diagnostics [9, 10].

In fact, vascular tissues exhibit sophisticated nonlinear and viscoelastic behaviors, including dynamic modulus, relaxation, and creep, that govern the deformation behavior of arteries under pulsatile pressure and the distribution of stress within the arterial microenvironment [14, 15, 16]. In the early stages of cardiovascular disease development, pathological remodeling at molecular and microstructural scales can perturb macroscopic mechanical phenotypes before overt changes become detectable by routine imaging or circulating biomarkers, supporting the value of mechanics‐informed early risk assessment [14]. Therefore, detecting multiple mechanical parameters of vascular lesions provides a more effective and direct approach for early warning of CVDs [14, 17]. In the mid‐to‐late stages of the disease, when atherosclerotic plaques are fully formed, the viscoelastic mechanical heterogeneity of different structural components, such as the fibrous cap (CF), fibrotic regions (IF), and necrotic core or lipid pool (LP), differs significantly, greatly influencing plaque rupture and lesion destabilization [15, 18, 19]. Thus, vascular mechanics provides a direct and quantitative perspective for evaluating the state and progression of lesions [14, 15].

Atomic force microscopy (AFM) enables nano‐ to microscale mechanical mapping of vascular tissues and has revealed pronounced intraplaque heterogeneity in experimental models and human samples [20]. Despite the advances in static mechanics of plaque, biological soft materials frequently exhibit power‐law rheology characteristics across time scales, reflecting the architecture changes of cytoplasm, cytoskeleton, and extracellular matrix (ECM) [21, 22, 23]. However, the understanding of multiscale, time‐dependent viscoelastic behavior across stages and plaque regions remains insufficiently characterized [18, 24]. Therefore, based on our previous studies, we aim to investigate the multiscale viscoelastic mechanical parameters of vascular tissue using AFM to quantify mechanical markers in the progression of atherosclerotic disease for diagnosis and risk assessment [18, 20].

In this study, we hypothesized that integrating biomechanical parameters of arterial lesions and plaque regions with traditional physiological and biochemical markers would improve the assessment of vascular lesion progression. To validate this hypothesis, we profiled vascular lesions using high‐resolution imaging, quantification of key inflammatory biomarkers and AFM‐based nano‐mechanical testing, to remodel the mechanobiological network of the correlations between imaging features, biochemical markers and mechanical parameters. We further developed machine learning models to integrate these features and predict vascular lesions and proposed a combined analysis approach that incorporates both vascular biological properties and mechanical properties. This integrated approach significantly improves the predictive and diagnostic accuracy for atherosclerosis. Additionally, we explored the biological mechanisms revealed by the association between inflammatory activity and mechanical plaque stability, aiming to identify likely mechanical biomarkers with translational potential for disease staging and non‐invasive diagnosis.

## 2 Results

### 2.1 Static Mechanical Behaviors

First, we examined the distinct structural components (Figure 1A) that arise during the development of atherosclerotic vascular lesions using Oil Red O, Masson, and von Kossa staining, as these features are critical for understanding their mechanical behavior. All tissue samples were subjected to serial cryosectioning. Slices containing intact arteries were selected for histological analysis, while adjacent slices were used for subsequent mechanical measurements. As shown in Figure 1B, multiple stains revealed no obvious morphological alterations at the early stages (0, 2, and 6 weeks). Oil Red O staining revealed the emergence of a necrotic core or LP in the mid‐to‐late stages (12 and 20 weeks), a structural hallmark of advanced atherosclerotic plaques. Masson staining highlighted extensive collagen accumulation around the plaque that is essential for maintaining plaque structural integrity; these dense collagen fiber characteristics were used to identify the CF and IF at late stages. Meanwhile, von Kossa staining revealed focal mineral deposition in late‐stage lesions, indicating the emergence of plaque calcification. These regional differences defined the microstructural basis for the mechanical heterogeneity observed in subsequent analyses.

**FIGURE 1:**
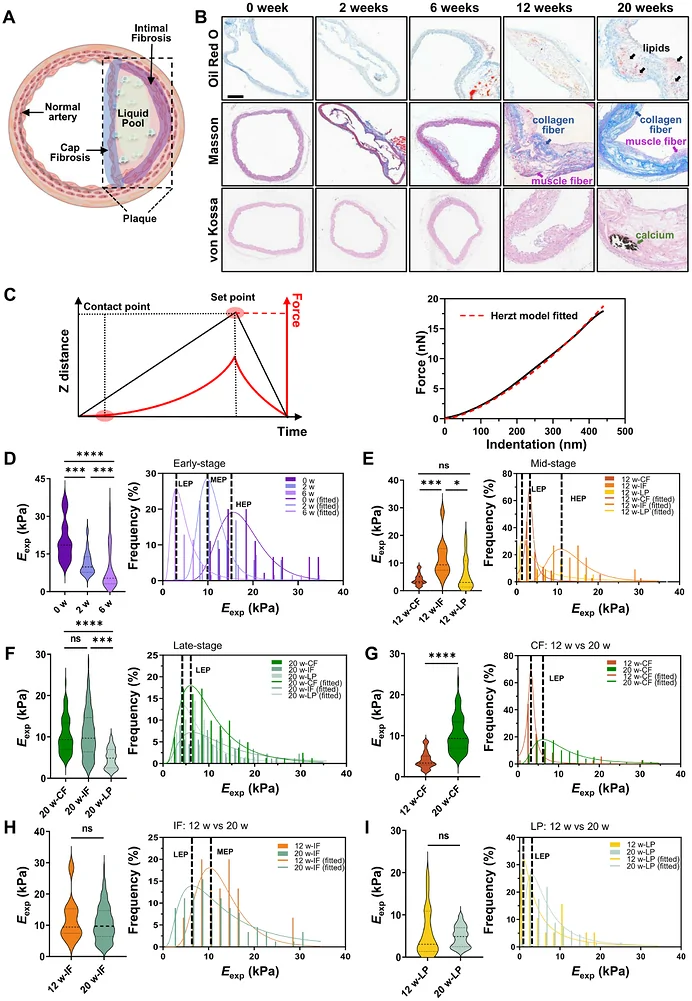
Stage‐ and region‐dependent stiffening during atherosclerotic lesions. (A) Schematic illustration of normal and atherosclerotic arteries showing cap fibrosis (CF), intimal fibrosis (IF), and lipid pool (LP). (B) Representative histological analysis of arteries at 0, 2, 6, 12, and 20 weeks, stained with Oil Red O (lipids), Masson (collagen fibers), and von Kossa (calcium). Scale bar, 200 µm; *n* = 3. (C) Schematic of AFM indentation and representative force–indentation curve fitted with the Hertz model to obtain the experimental elastic modulus (*E_exp_*). (D–F) *E_exp_* and corresponding distributions at early (0–6 weeks), mid (12 weeks), and late (20 weeks) stages. (G–I) *E_exp_* and corresponding distributions in CF, IF, and LP regions at 12 and 20 weeks. LEP, MEP, and HEP denote low‐, mid‐, and high‐elasticity peaks, respectively. Detailed sample numbers for each mechanical parameter are provided in Table S6. Statistical analysis was performed using the Kruskal‐Wallis nonparametric test followed by the two‐stage step‐up method of Benjamini, Krieger, and Yekutieli distribution was statistically analyzed using the Kolmogorov‐Smirnov (K‐S) test. ^*^ *p* < 0.05, ^***^ *p* < 0.001, ^****^ *p* < 0.0001, ns: no significance.

The histopathological staining revealed a time‐dependent progression from mild intimal expansion at 0–6 weeks to well‐stratified atherosclerotic plaques at 12–20 weeks, consisting of CF, IF, and LP. Based on these histological features, we stratified samples according to disease stage. Samples from the early stage were analyzed as intact arterial tissue, whereas in the mid‐to‐late stage, when the distinct plaque substructures became apparent, three distinct regions (CF, IF, and LP) within the plaques were analyzed separately.

To further dissect the mechanical properties of heterogeneous plaques, we performed AFM‐based local mechanical characterization co‐registered with histological metrics, revealing that the arterial elastic modulus changes dynamically throughout disease progression. The static mechanical testing, as schematized in Figure 1C, was performed at randomly chosen locations within the same anatomical region on cryo‐sections of 15 µm. Given the relatively large tissue area of the artery or plaque (≥15,000 µm^2^), the significant distance between individual measured locations (∼200 µm), and the small analyzed area of each location, we consider the measurement at each location to represent a complete mechanical assessment equivalent to one sample, as in our previously reported methodology [18]. Therefore, we believe that our methodology sufficiently captures the diversity of mechanical characteristics of different plaque regions. A relatively large micron‐scale probe (tip radius = 10 µm) was employed to characterize the average mechanical properties of various periods (0, 2, 6, 12, 20 weeks) and components (CF, IF, and LP), rather than focusing on individual microstructural constituents. During measurements, the applied force at all locations was tightly controlled at 20 nN. Our examination revealed a wide spectrum of static mechanical properties across distinct atherosclerotic stages and regions, spanning a range from 1.32 to 37.48 kPa (Table S1). The static modulus was highest at week 0 (18.536 kPa), decreased to a minimum by week 6 (Figure 1D), remained relatively low through 12 weeks (5.897 kPa) (Figure 1E), and then increased in 20 weeks (9.906 kPa) (Figure 1F) ‐although it did not return to the initial level. In the mid‐to‐late stages, when plaques displayed regional heterogeneity, the changes in modulus were most evident in the CF region (Figure 1G). The elastic modulus across regions showed that LP consistently exhibited the lowest and most similar modulus at all time points, whereas the IF 1515region had a higher and relatively consistent modulus. In contrast, the CF region displayed a progressive increase from 12 weeks (6.15 kPa) to 20 weeks (12.06 kPa), representing nearly a twofold rise. However, during these stages, the elastic modulus reliably distinguished neither between CF and LP regions at 12 weeks (Figure 1E), nor between CF and IF regions at 20 weeks (Figure 1F). Similarly, the elastic modulus failed to distinguish IF and LP regions during 12 to 20 weeks (Figure 1H,I). Additionally, we revealed considerable variability in stiffness values across stages and regions, as indicated by the coefficients of variation (CV) (Table S1). Consequently, relying solely on the elastic modulus is insufficient for accurately and efficiently discriminating the mechanical properties of atherosclerotic lesions. The pronounced heterogeneity in local mechanics mirrors the mixed histological composition of plaques, arising from regional variations in cell density, lipid accumulation, and ECM organization [20, 25, 26]. These stiffness differences are therefore attributable to compositional and structural contrasts, particularly the enrichment of collagen in fibrous domains [20, 23, 25, 26, 27].

### 2.2 Dynamic Mechanical Responses

To compensate for the limitations of elastic modulus, we next interrogated the viscoelastic behaviors (Dynamic creep displacement and relaxation) of atherosclerotic lesions, aiming to yield comprehensive insights into their underlying mechanical nature. Dynamic creep displacement (*D_creep_*) is defined as the total displacement of the probe during creep under a constant load, reflecting the time‐dependent deformation of the sample. The relaxation behavior was characterized by the half‐relaxation time (*τ_1/2_*), defined as the time required for the measured force to decrease to 50% of its change value following indentation. Also guided by our histological findings (Figure 1B), we used the same approach, but the applied force was strictly maintained at 20 nN for 10 s during the creep (Figure S1), and the Z‐sensor movement was limited to 1 µm for 10 s at all locations during the relaxation (Figure S2) [20, 28].

Dynamic creep displacement (*D_creep_*) exhibited pronounced spatiotemporal heterogeneity during lesion progression (Table S2). After an initial decrease at early time stages, *D_creep_* increased with advancing disease (Figure 2A). Once discrete plaque regions emerged, a consistent mechanical gradient was observed under identical loading, in which LP exhibited the largest *D_creep_*, whereas CF and IF clustered at substantially lower levels (Figure 2C). Notably, LP at 20 weeks (717.3 nm) was nearly twice that observed at 12 weeks (400.3 nm), indicating a pronounced time‐dependent amplification of viscoelastic deformation within lipid‐rich domains. Such escalation in time‐dependent deformation is consistent with prior evidence that lipid‐rich regions are mechanically distinct (generally softer than collagen‐rich fibrous regions) and contribute substantially to intraplaque mechanical heterogeneity [18, 20]. Additionally, the late period exhibits a multimodal distribution from low‐creep peak (LCP, ∼100 nm) to high‐creep peak (HCP, ∼1000 nm) at 20 weeks (Figure 2B,D). The pronounced rightward shifts in the *D_creep_* distributions across components further underscore these mechanical disparities.

**FIGURE 2:**
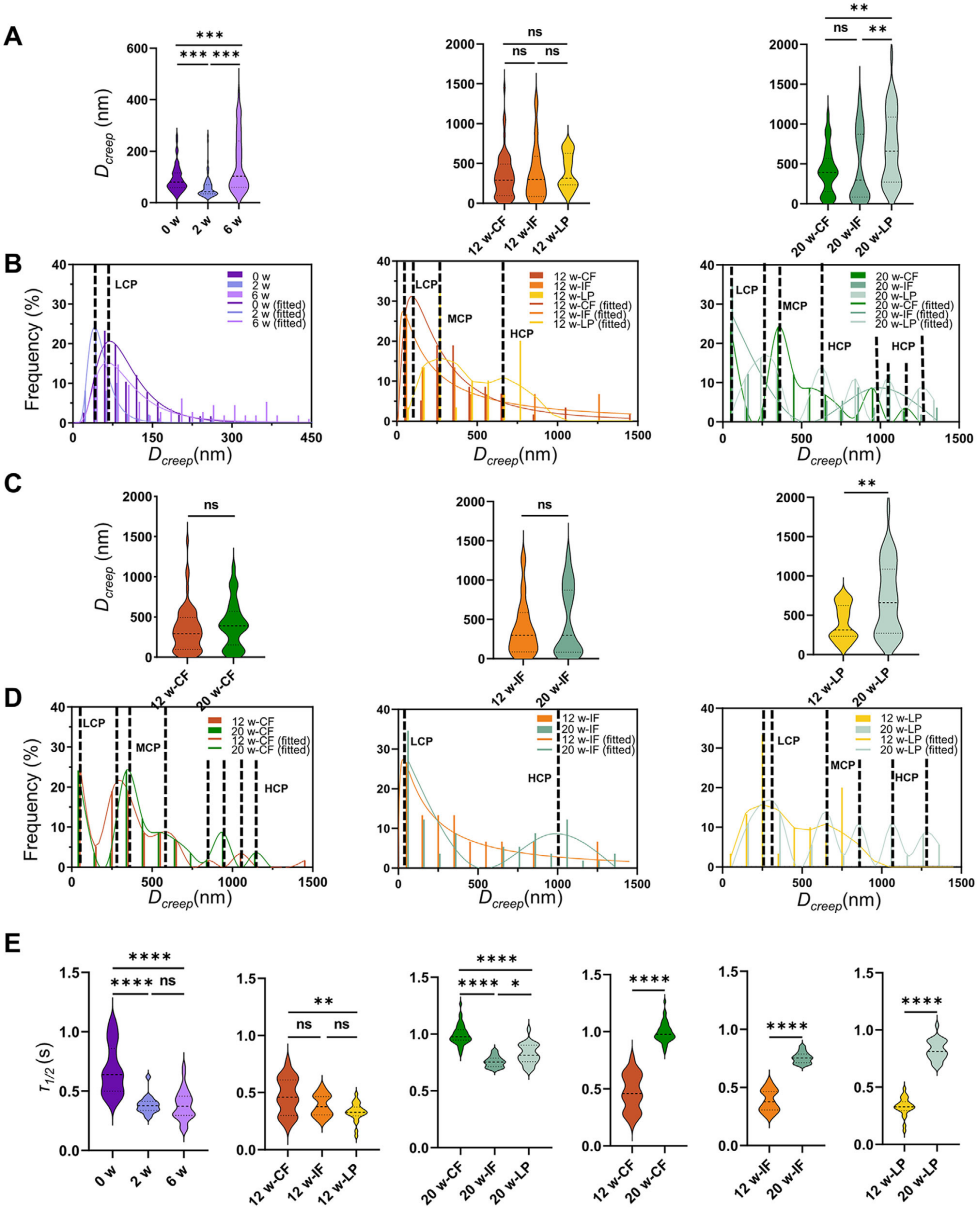
Creep and relaxation dynamics reveal stage‐ and region‐dependent viscoelastic remodeling during atherosclerotic progression. (A) *D_creep_* at early (0–6 weeks), mid (12 weeks), and late (20 weeks) stages. (B) Corresponding distributions of *D_creep_* at different disease stages. LCP, MCP, and HCP denote low‐, mid‐, and high‐creep peaks, respectively. (C) Comparison of *D_creep_* in CF, IF, and LP between 12 and 20 weeks. (D) Corresponding distributions of *D_creep_* in CF, IF, and LP. (E) Half‐relaxation time (*τ_1_/_2_*) at different disease stages and plaque regions. Detailed sample numbers for each mechanical parameter are provided in Table S6. Group comparisons were performed using the Kruskal–Wallis nonparametric test with the two‐stage step‐up method of Benjamini, Krieger, and Yekutieli; distributions were compared using the Kolmogorov–Smirnov test. ^**^ *p* < 0.01, ^***^ *p* < 0.001; ns, no significance.

In addition to creep compliance measurements, we quantified atherosclerotic lesions' viscoelasticity using relaxation tests. The relaxation results of the tissues were also characterized (Table S3). The vascular relaxation half‐time (*τ_1/2_*) decreased gradually from 0.6424 s at 0 weeks to ∼0.38 s, and remained constant through 6 weeks (Figure 2E), indicating a rapid transition from slow to fast relaxation in the early phase of plaque formation. Moreover, the overall relaxation behavior at 12 weeks was similar to that at 6 weeks; CF and IF displayed comparable *τ_1/2_*, whereas LP showed a significantly shorter *τ_1/2_*, consistent with a highly fluid‐like and weakly constrained microenvironment in lipid‐rich regions. However, with the emergence of region‐specific plaque heterogeneity, CF exhibited the longest *τ_1/2_* among components. This regional hierarchy persisted at 20 weeks, at which point *τ_1/2_* values exceeded earlier time points by approximately 1.5–3‐fold, indicating a marked late‐stage prolongation of viscoelastic relaxation. However, the IF region at 20 weeks showed the shortest *τ_1/2_*, and LP adopted an intermediate behavior. Longitudinal comparisons within each region further revealed that *τ_1/2_* increased from 12 weeks to 20 weeks.

### 2.3 Two‐Stage Power‐Law Rheology

Biological tissues exhibit complex viscoelastic behaviors arising from the coexistence of solid‐like elasticity and fluid‐like viscosity. Under dynamic mechanical loading, soft biological materials display time‐dependent responses such as creep and relaxation. Unlike classical viscoelastic materials characterized by discrete exponential relaxation, many biological tissues follow a power‐law rheological behavior, in which deformation and mechanical modulus exhibit scale‐free temporal dependence. In this framework, the power‐law exponent α reflects the fluidity of the material, where lower α values indicate more solid‐like behavior and higher values correspond to enhanced viscous dissipation. A precise understanding of atherosclerotic plaque mechanics, especially their time‐dependent viscoelasticity, is essential for predicting disease trajectory and rupture risk [18]. To address this challenge, we employed AFM‐based microrheology to characterize the time‐dependent creep responses of individual plaque components. AFM directly records the force and indentation histories rather than spatially resolved stress and strain fields. Therefore, instead of calculating local stress and strain separately, we derived an effective indentation creep compliance, *J_ind_* (*t*), from the measured force–indentation response using the spherical Hertz contact relation combined with the linear‐viscoelastic correspondence principle. Under the constant‐force condition, the progressive increase in indentation depth reflected the time‐dependent compliance of the tissue. The resulting *J*_ind_(*t*)was plotted in log–log coordinates to quantify the short‐ and long‐timescale power‐law exponents. In our previous work [18, 21, 29, 30, 31], we introduced power‐law rheology to assess subtle viscoelastic changes in different plaque components. As the indentation depth increased during the creep process, we observed a corresponding time‐dependent rise in creep compliance, characterized by short‐time (*α_short_*: 0.01‐0.1 s) and long‐time (*α_long_*: 1–10 s) power‐law exponents [22, 29] (Figure S3).

As illustrated in Figure 3A–F, we revealed distinct rheological behaviors across stages and regions, delineating a wide viscoelastic spectrum within atherosclerotic lesions. At shorter timescale, *α_short_* decreased from 0.4139 (0 week) to 0.3870 (2 weeks), and then increased to 0.5224 (6 weeks) at the early stage (Figure 3A; Table S4). At 12 weeks, CF exhibited the highest *α_short_* (Table S4, 12w‐CF = 0.7540), whereas CF and IF showed similar values (Figure 3B). But at 20 weeks, the LP region displayed the highest *α_short_*, IF the lowest, and CF an intermediate distribution (Figure 3C). Furthermore, a bimodal distribution in rheology was evident during 20 weeks at LP regions (Figure 3F), with one rheology peak at other stages and regions (Figure 3D,E). The high‐rheology peak (HRP, = 0.8) for 20w‐LP was likely to encapsulate the viscoelastic property of the intracellular matrix (ICM) and ECM networks. In contrast, the low‐rheology peak (LRP) that was around 0.4 appeared to reflect the dynamics of compressed ICM in the absence of ECM at the early stage and in the lipid‐rich regions. The rheological behavior of plaque components at shorter timescales was predominantly influenced by the relaxation and reorganization of cytoplasmic structures, alongside rearrangements within the cytoskeleton and ECM [18].

**FIGURE 3:**
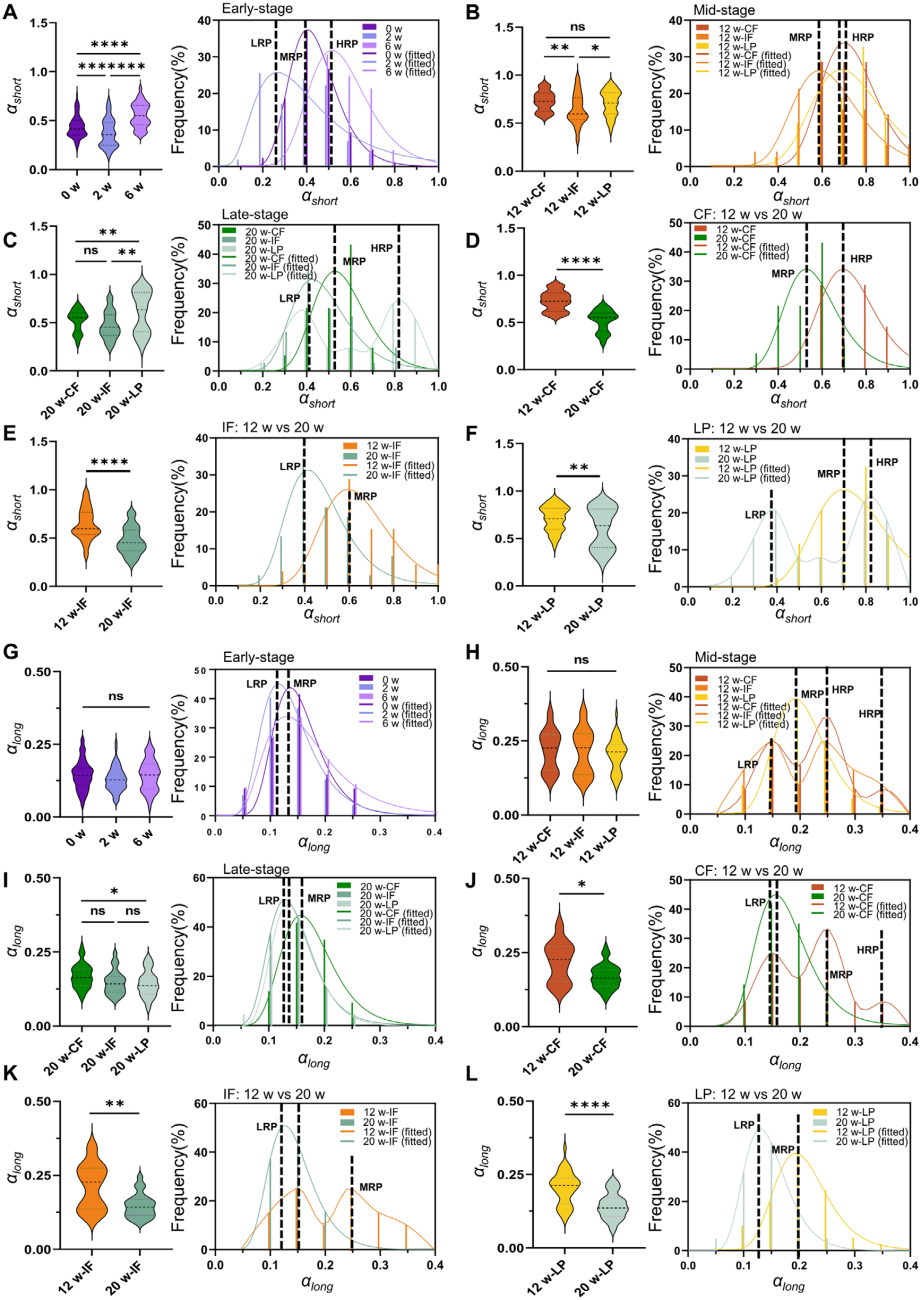
Two‐stage power‐law rheology reveals stage‐ and region‐specific fast and slow compliance dynamics during atherosclerotic progression. (A–C) Short‐time power‐law exponent (*α_short_*, 0.01‐0.1 s) and corresponding distribution profiles at early (A), mid (B), and late (C) stages. (D–F) Region‐specific comparison of αshort in CF, IF, and LP between 12 and 20 weeks. (G–I) Long‐time power‐law exponent (*α_long_*, 1–10 s) and corresponding distribution profiles at early (G), mid (H), and late (I) stages. (J‐L) Region‐specific comparison of *α_long_* in CF, IF, and LP between 12 and 20 weeks. LRP, MRP, and HRP denote low‐, mid‐, and high‐rheology peaks identified from Lorentz‐fitted distributions. Detailed sample numbers for each parameter are provided in Table S6. Group comparisons were performed using the Kruskal–Wallis nonparametric test with the two‐stage step‐up method of Benjamini, Krieger, and Yekutieli; distributions were compared using the Kolmogorov–Smirnov test. ^*^ *p* < 0.05, ^**^ *p* < 0.01, ^***^ *p* < 0.001, ^****^ *p* < 0.0001; ns, no significance.

As illustrated in Figure 3G–L, *α_long_* shows no significant differences in early‐to‐mid stage (0–12 weeks; Figure 3G–I) except between 20w‐CF and 20w‐LP. By 12 weeks, CF exhibited the highest *α_long_* (12w‐CF = 0.2190), establishing a pronounced long‐timescale relaxation gradient. In comparing *α_long_* distributions, we noted a multimodal distribution in the 12w‐CF and ‐IF region (LRP = 0.15, MRP = 0.25, and HRP = 0.35) (Figure 3H), indicating varying degrees of viscoelasticity. The distinct *α_long_* distributions reflected the unique viscoelastic properties of each plaque region. The LRP of IF was more likely to capture subtle viscoelastic behavior of collagen content that was rich in the CF region, generating a smaller exponent. Conversely, the rheological property of the lipid‐rich region LP can be effectively described using a single peak with a high exponent (high‐rheology peak (HRP) ∼ 0.8). Although power‐law rheology has been extensively studied in diverse biological systems, including epithelial monolayers [32], single cells [33], cytoplasm [34], and nucleus [35], studies focusing on tissue‐scale power‐law rheology are scarce. Our study delved into the viscoelastic behavior of different plaque components, uncovering a universal two‐stage power‐law behavior in their dynamic creep response. Distinct power‐law rheology variations across plaque regions provided a robust approach to assess viscoelastic heterogeneity and gradients, offering insights into atherosclerotic plaque states and vulnerability.

### 2.4 Multiscale Mechanical Heterogeneity Across Stages and Regions

Building upon our previous foundational work, we further extend the Hybrid Hierarchical theory–Microrheology (HHM) framework by refining the second and third hierarchies to explicitly incorporate the full cytoskeleton and the transverse expansion of the tissue ECM (Figure S4) [18, 21, 29, 30]. The creep compliance of each hierarchy is then determined as follows:

Hierarchy 1 (*J_1_*): The cytoplasm is modeled as an elastic network of stiffness (elastic stiffness *E_1_*) embedded in a viscous cytosol (viscosity *η*), yielding the baseline creep response.

Hierarchy 2 (*J_2_*): Additional complexity from cellular structures in the cellular cytoskeleton is captured by an additional series of effective springs (stiffness *E_2_*).

Hierarchy 3 (*J_3_*): The surrounding ECM is represented by series‐connected spring elements that account for transverse ECM expansion, with effective stiffness *E_3_*.

The corresponding creep compliances are

(1) $$J_{1}=\frac{-1+\sqrt{1+\frac{4t}{\tau }}}{2E_{1}}$$

(2) $$J_{2}=\frac{-1+\sqrt{1+4E_{2}J_{1}}}{2E_{2}}$$

(3) $$J_{3}=\frac{-1+\sqrt{1+4E_{3}J_{2}}}{2E_{3}}$$

respectively. The cytoplasmic viscosity *η* = *τ* × *E_1_*.

To dissect how intracellular and extracellular components contribute to plaque mechanics, we employed this theoretical model and quantified the hierarchical stiffness parameters *E_1_*, *η*, *E_2_*, and *E_3_* across developmental stages and plaque regions. First of all, the cytoplasmic stiffness (*E_1_*) increased progressively from 0 to 6 weeks and showed clear regional separation at 12 and 20 weeks, with CF displaying the highest values and LP the lowest (Figure 4A). As for the cytoplasmic viscosity (*η*), the cell also exhibited the increased behavior over time and remained significantly higher in CF compared with IF and LP at both 12 and 20 weeks (Figure 4C), consistent with enhanced dissipative behavior in fibrotic regions. Regarding cytoskeletal stiffness (*E_2_*), cells exhibited a similar trend but showed an even sharper contrast among regions (Figure 4B), indicating robust cytoskeletal reinforcement in CF and pronounced degradation in IF and LP. Finally, we analyzed the transverse expansion of ECM stiffness (*E_3_*) at the tissue level, which represents the most macroscopic hierarchy. The *E_3_* measurements demonstrated the strongest regional separation (Figure 4D), with CF showing markedly elevated values that further increased at 20 weeks, reflecting progressive ECM compaction and collagen crosslinking.

**FIGURE 4:**
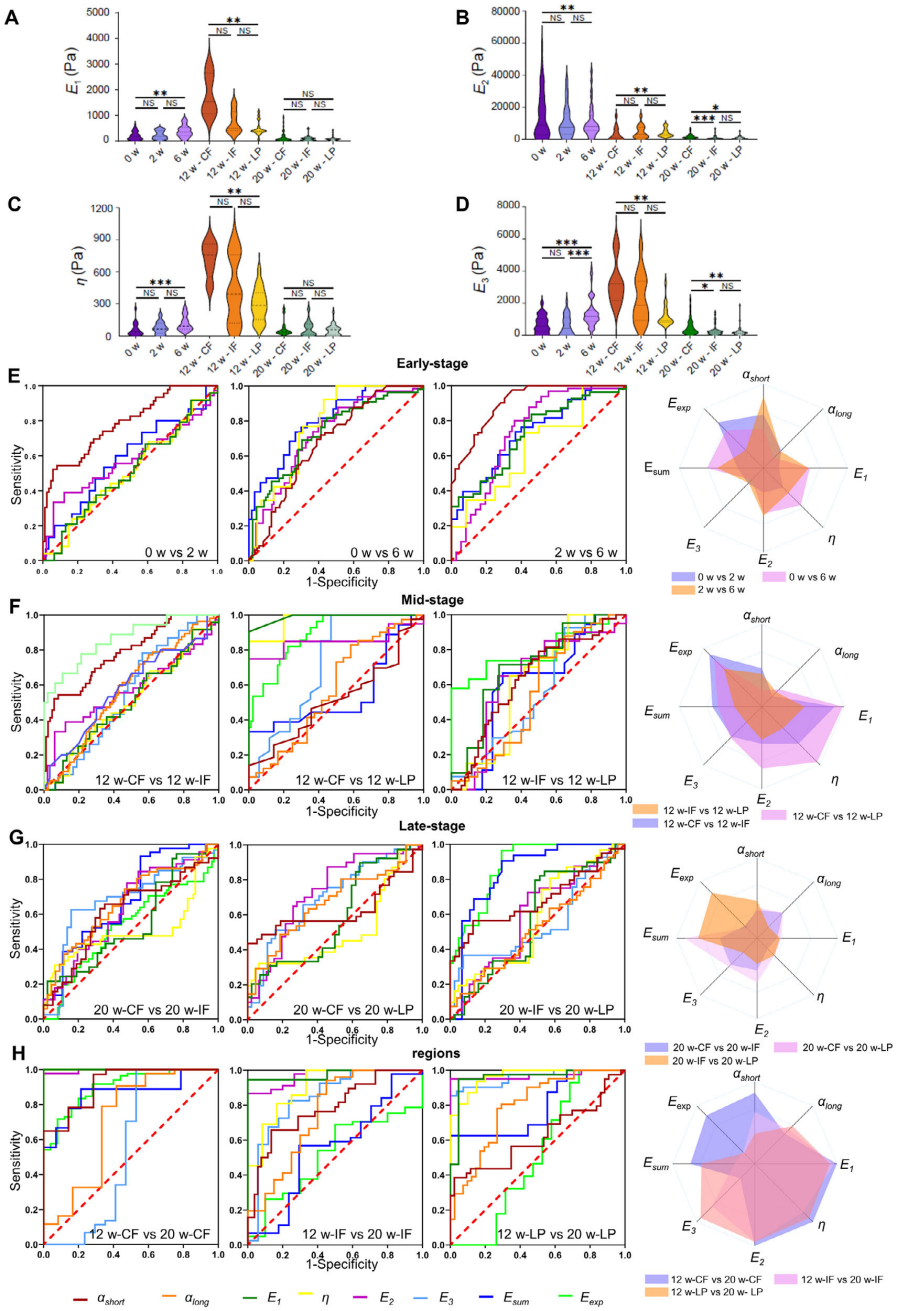
Hierarchical viscoelastic parameters and their diagnostic performance across disease stages and plaque regions. (A–D) Violin plots of HHM‐derived mechanical parameters, including cytoplasmic stiffness (*E_1_*, A), effective cytoplasmic viscosity (*E_2_*, B), cytoskeletal stiffness (*η*, C), and extracellular matrix transverse stiffness (*E_3_*, D), across atherosclerotic stages and plaque regions. (E–G) ROC curves and corresponding AUC radar plots evaluating the discriminative performance of hierarchical mechanical parameters and power‐law exponents at early (E), mid (F), and late (G) stages. (H) ROC analysis and AUC radar plots for region‐specific discrimination among CF, IF, and LP at mid and late stages. Detailed sample numbers for each mechanical parameter are provided in the Methods and Table S6. Group comparisons were performed using the Kruskal–Wallis nonparametric test with the two‐stage step‐up method of Benjamini, Krieger, and Yekutieli. ^*^ *p* < 0.05, ^**^ *p* < 0.01, ^***^ *p* < 0.001; ns, no significance.

Up to now, our study has introduced eight distinct mechanical parameters, each varying across atherosclerotic lesions, aimed at enhancing diagnosis. To evaluate the efficacy of these mechanical indexes, we utilize receiver operating characteristic (ROC) curves, calculating Area Under Curve (AUC) to determine their discriminative capacity. ROC analyses revealed that *E_3_*, *E_sum,_* and *E_exp_* consistently achieved the highest discriminative power across stages (Figure 4E–H). Early‐stage discrimination was modest, whereas mid‐ and late‐stage comparisons yielded AUCs approaching 0.9, particularly for CF vs LP. IF showed intermediate profiles, resulting in weaker separability, reflecting its transitional mechanical phenotype. Radar plots further highlighted distinct multiscale mechanical signatures, with CF characterized by uniformly high stiffness across all hierarchies, LP by uniformly low stiffness and high fluidization, and IF by partial weakening of cytoskeletal and ECM parameters accompanied by elevated *α_short_*.

The ROC analysis further underscores that ECM mechanics—not cytoplasmic or cytoskeletal contributions—serve as the most sensitive markers of plaque identity and stage. This is consistent with classical theories of plaque vulnerability, where fibrous cap strength and ECM integrity determine rupture risk. The radar plots illustrate a multiscale mechanical gradient, with a stiff, slowly relaxing CF overlying a softer, fast‐relaxing IF and an even softer LP. Such hierarchical gradients are predicted to amplify shear and tensile stresses at material boundaries under pulsatile loading, providing a mechanistic explanation for localized vulnerability in advanced plaques [19].

The observed spatial and temporal heterogeneity in vascular creep necessitates a theoretical framework that links microscale constituents to macroscale mechanics. Our self‐similar hierarchical model resolves this by expressing the tissue elastic modulus (*E_sum_*) as the sum of elastic contributions from the cytoplasmic, cellular, and extracellular levels: *E_sum_* = *E_1_* + *E_2_* + *E_3_*. To assess model fidelity, we compared this theoretically derived modulus (*E_sum_*) with the experimentally measured modulus (*E_exp_*) at matched locations and demonstrated excellent agreement (Figure S5). This result provides strong experimental validation of the model's robustness and affirms its ability to accurately represent the measured mechanical landscape of atherosclerotic plaque states and vulnerability.

### 2.5 Relationship Between Biological Markers and Biomechanical Properties

Although our study delineated the distinct evolution of elastic modulus and viscoelastic properties during atherosclerotic lesion progression, the translational potential of these mechanical parameters hinges on their consistency with established clinical diagnostic approaches. To this end, we first benchmarked our biomechanical indices against conventional diagnostic pillars, namely morphological assessment (Figure S6A–C), blood lipid profiling (Figure S6D), and inflammatory factors. The qRT‐PCR analyses revealed strong temporal regulation of inflammatory mediators (Figure 5A). The expression levels of *IL‐6*, *IL‐1β*, and *TNF‐α* remained low at 0–2 weeks but increased sharply at 6–12 weeks, marking the peak of inflammatory activation. By 20 weeks, their expression declined yet remained elevated relative to baseline. In contrast, the anti‐inflammatory cytokine *IL‐10* [36] increased modestly at mid‐stage and reached its highest levels at 20 weeks, indicating a late‐stage shift toward reparative signaling. Macrophages are key regulators of atherosclerosis‐ either promoting plaque progression and vulnerability or contributing to inflammation resolution and tissue remodeling [37, 38, 39, 40]. So we next analyzed the polarization states of macrophages during atherosclerotic lesion development (Figure 5B; Figure S7). Immunohistochemistry showed minimal CD68^+^ macrophage infiltration at 0–2 weeks, followed by extensive accumulation at 6–12 weeks in ApoE^−^/^−^ mice. At 20 weeks, macrophages remained abundant but displayed a more peripheral distribution, consistent with fibrous cap development. Immunofluorescence imaging demonstrated a clear polarization trajectory (Figure 5C). M1 macrophages dominated at 6–12 weeks, coinciding with the peak of pro‐inflammatory cytokines. At 20 weeks, M2 macrophages substantially increased, reflecting a transition toward tissue remodeling and fibrosis. Then, we rigorously evaluated the association between our multiscale mechanical measurements and conventional metrics of disease severity (Figure 5D–F) [41]. The correlation networks illustrated distinct stage‐dependent interaction patterns. Across all time points, pro‐inflammatory cytokines (*IL‐6*, *IL‐1β*, *TNF‐α*) showed strong positive correlations with mechanical softening markers such as αshort, *D_creep_*, and reduced *E_1_*/*E_2_*, whereas *IL‐10* exhibited opposite correlations.

**FIGURE 5:**
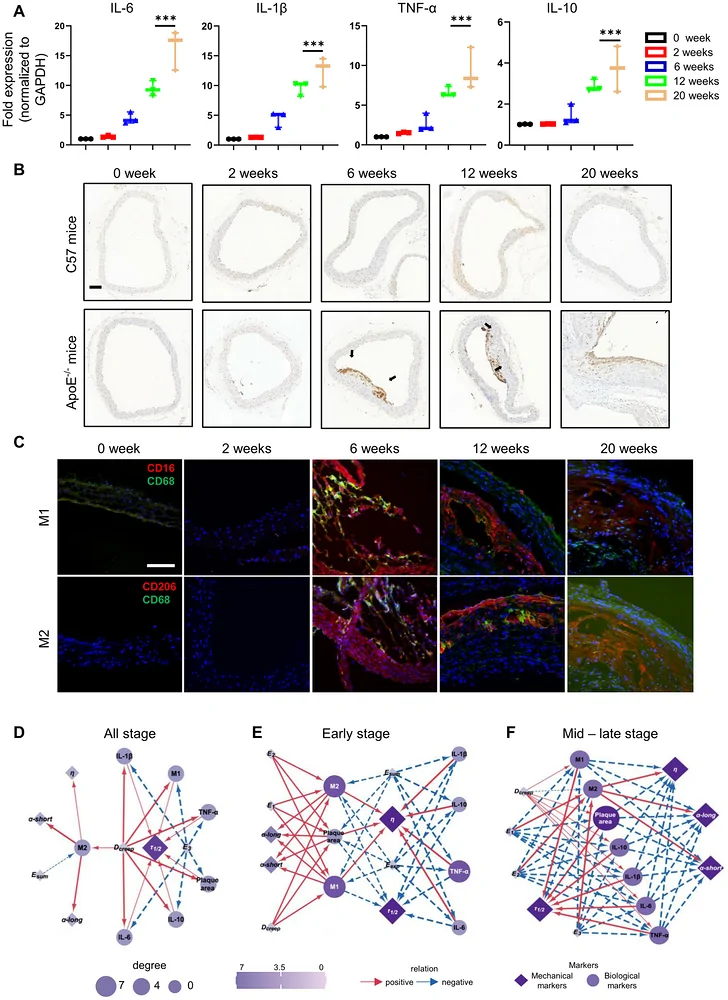
Mechanobiological network remodeling based on the trends of inflammatory factors, macrophage polarization, and their correlation with mechanical parameters changes during atherosclerotic plaque progression. (A) Quantification of the relative expression level of pro‐inflammatory *IL‐6*, *IL‐1β*, *TNF‐α*, and anti‐inflammatory *IL‐10* in artery tissue across disease stages (*n* = 5 each). (B) Representative immunohistochemical staining of CD68^+^ macrophages in artery tissue across disease stages (*n* = 3 each). Scale bar, 100 µm. (C) Immunofluorescence images showing dynamic macrophage polarization within arterial or plaque tissue during atherosclerotic progression. M1 macrophages (CD16^+^/CD68^+^, top row) dominate at 6–12 weeks, consistent with inflammatory activation and tissue degradation. M2 macrophages (CD206^+^/CD68^+^, bottom row) increase prominently at 20 weeks, aligning with ECM deposition and structural stabilization in advanced lesions (*n* = 3 each). Scale bar, 100 µm. (D–F) Integrated mechanobiological correlation network illustrating global interactions between mechanical parameters (diamonds) and biological markers (circles) in all stages (D), early (E), and mid‐late (F) stages. The edges represent positive (red) or negative (blue) correlations; node size reflects degree centrality. The edge connecting a pair of nodes indicates the coincidence of the corresponding features, with its thickness proportional to the coincidence rate; the edges are displayed if the rate exceeds 5%. Data in A are shown as mean SD.

Further detailed analysis also reflected similar but slightly different results. During early stages, fast‐relaxation markers were the most strongly connected to inflammatory mediators, suggesting that cytoplasmic fluidization accompanies inflammatory initiation. In mid‐to‐late stages, ECM‐level parameters (*E_3_*, *E_exp_*, *E_sum_*) became the dominant correlated nodes and aligned positively with *IL‐10* and M2 markers, indicating that mechanical stiffening and matrix consolidation are tightly coupled to reparative immune responses. The sharp rise in *IL‐6*, *IL‐1β*, and *TNF‐α* at 6–12 weeks, along with robust macrophage infiltration, indicates that plaque expansion is initially governed by strong inflammatory activation. Their positive correlation with softening markers (higher *α_short_*, greater *D_creep_*, reduced *E_1_*/*E_2_*) suggests that inflammatory signaling was associated with cytoskeletal disruption and ECM degradation, consistent with known roles of M1 macrophages in promoting tissue destabilization. The late‐stage increase in *IL‐10* and M2 macrophages coincides with rising ECM stiffness markers (*E_3_*, *E_exp_*, *E_sum_*), implying a shift toward fibrosis and structural reinforcement. This mechanobiological transition supports a model in which early inflammation fluidizes tissue, while late reparative was consistent with consolidating the ECM, generating the stiff, slowly relaxing fibrous cap characteristic of advanced plaques [9, 20, 21, 27, 42, 43, 44]. The correlation networks reveal that mechanical and inflammatory processes are not independent but evolve as integrated modules: (1) Early stage: “Inflammation and cytoplasmic fluidization” module dominates; (2) Mid stage: “Inflammation and cytoskeletal degradation” becomes prominent; (3) Late stage: “Reparative immunity and ECM stiffening” emerges as the dominant module. This shifting mechanobiological landscape explains the emergence of heterogeneous plaque regions and provides a mechanistic basis for stage‐specific vulnerability. These findings demonstrate that inflammatory cues and viscoelastic remodeling are tightly coupled across disease development. The identification of stage‐specific mechanical–biological signatures provides a rationale for integrating mechanical markers into diagnostic frameworks and suggests new mechanobiological targets for early intervention [41].

### 2.6 Multiparametric‐Based Machine‐Learning Model for Assessing Atherosclerotic Lesions

While the role of ECM elastic modulus in disease has been extensively studied, its utility as a standalone metric has shown limitations during atherosclerosis progression. Therefore, we evaluated whether mechanical markers could be used to classify disease stage and plaque location. Using 218 measurements of atherosclerotic lesion mechanics, we trained an Ensemble softmax neural‐network model (EnsNN) to compare single‐feature classification (elastic modulus) with multi‐feature classification of disease progression (Figure 6A).

**FIGURE 6:**
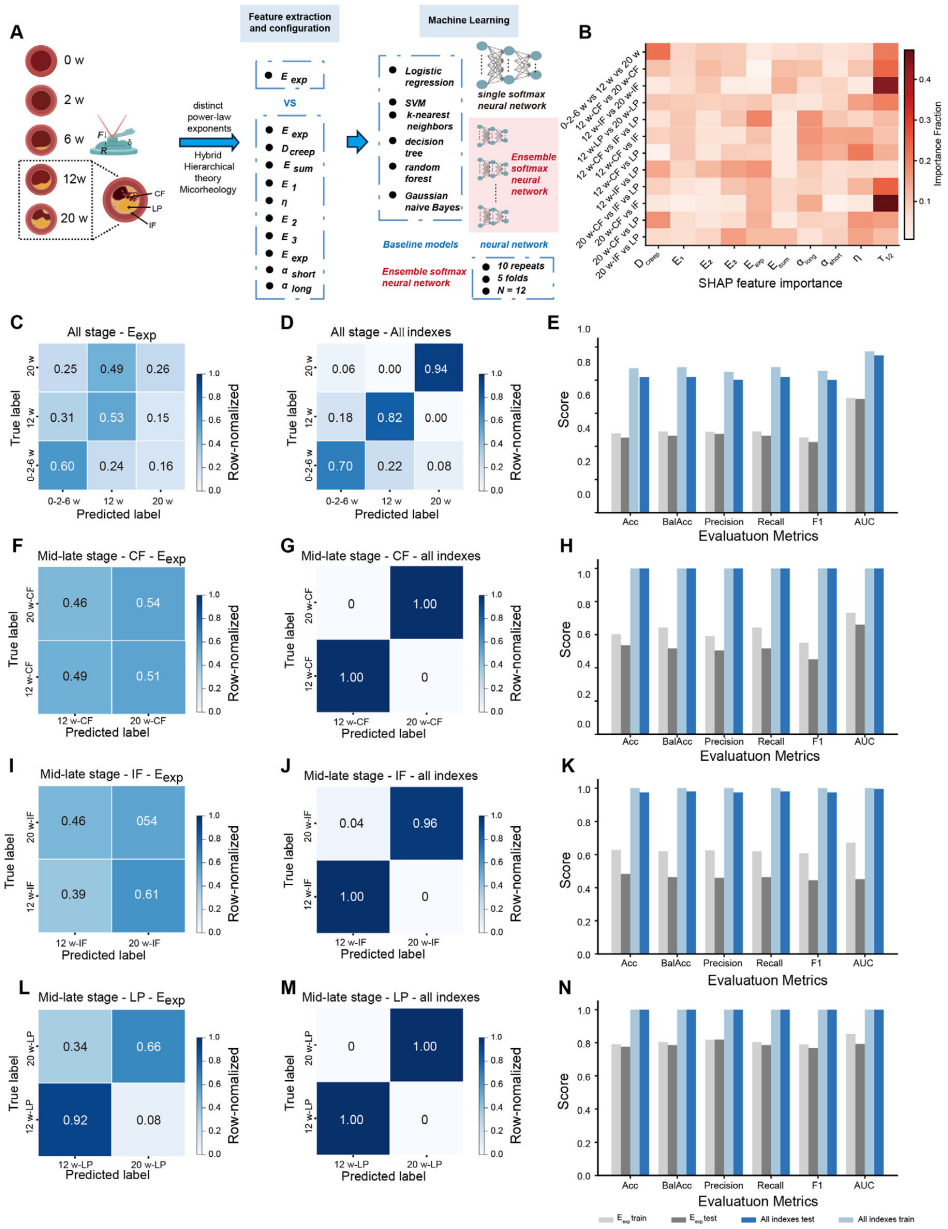
Multiscale viscoelastic‐parameter‐based ensemble neural‐network learning for characterization of atherosclerotic progression and plaque heterogeneity. (A) Workflow of mechanical‐marker extraction, feature‐set construction, baseline‐model comparison, and ensemble softmax neural‐network classification. (B) SHAP importance map for the full‐parameter EnsNN model, indicating that classification relied on multiple complementary viscoelastic markers rather than *E*_exp_ alone. (C–N) Representative held‐out cross‐validated classification results comparing *E*_exp_alone and full‐parameter models. Row‐normalized confusion matrices and evaluation metrics are shown for all‐stage classification (C–E) and 12‐ versus 20‐week regional classification in CF (F–H), IF (I–K), and LP (L–N) regions. For each task, row‐normalized held‐out confusion matrices compare the *E*_exp_ alone model with the all‐index model, and bar plots summarize accuracy, balanced accuracy, precision, recall, F1‐score, and AUC. Gray bars indicate the *E*_exp_ alone model, whereas blue bars indicate the all‐index model; light and dark shades denote training and held‐out test performance, respectively. The full multiscale mechanical‐parameter set consistently improved class separation and predictive performance, supporting its added value for identifying disease progression and plaque mechanical heterogeneity.

To evaluate whether a neural‐network classifier was necessary, we compared the EnsNN with several baseline classifiers, including logistic regression, SVM, k‐nearest neighbors, decision tree, random forest, and Gaussian naive Bayes. All models were evaluated using the same repeated stratified five‐fold cross‐validation procedure. As summarized in Figure S8, the EnsNN achieved the best overall balance among the tested classifiers, with the highest mean held‐out macro‐F1 and the best mean task‐wise rank. Across all classification tasks, the all‐index EnsNN reached a mean held‐out accuracy of 0.885 and a mean macro‐F1 of 0.877. While linear SVM, random forest, RBF‐SVM, and logistic regression remained competitive in individual tasks, they did not simultaneously match the EnsNN in average predictive performance and ranking consistency. The EnsNN achieved the best overall trade‐off between predictive performance and stability across tasks. Across all classification tasks, it achieved a mean held‐out test accuracy of 0.882 and a mean held‐out macro‐F1 of 0.872. It also showed the best overall mean rank and appeared within the top two models in 75.0% of the tasks (Figure S8). Compared with the single softmax NN, the EnsNN showed higher mean test macro‐F1, lower repeat‐to‐repeat variability, and a smaller train‐test performance gap. This suggests that ensembling reduced the sensitivity of the neural‐network classifier to random initialization and improved the stability of its predictions. Importantly, simpler classifiers still performed best in some individual tasks. Therefore, the ensemble NN was not selected because neural networks were assumed to be universally superior, but because it provided the most favorable overall balance among held‐out predictive performance, class‐balanced metrics, stability, and generalization. We further examined the effect of the number of subnetworks in the ensemble. Increasing the ensemble size from N = 1 to N = 3 improved the mean held‐out test macro‐F1, after which performance reached a plateau. Although N = 3 already achieved near‐saturated mean performance, the N = 12 ensemble showed the lowest repeat‐to‐repeat variability. Therefore, N = 12 was selected as a conservative setting to improve robustness rather than to maximize a single split‐dependent accuracy value (Figure S9). In addition, the all‐index EnsNN markedly outperformed the *E*_exp_‐only model, which achieved only 0.637 mean held‐out accuracy and 0.607 mean macro‐F1 across the 12 tasks. Incorporating the full mechanical‐marker panel increased accuracy and macro‐F1 by 0.248 and 0.270, respectively, with macro‐F1 improved in 11 of 12 tasks, supporting the added diagnostic value of multiscale viscoelastic parameters beyond apparent elastic modulus alone.

To visualize classification errors, confusion matrices were generated from held‐out test predictions only. These matrices confirmed that the all‐index model reduced misclassification in most tasks compared with the *E_exp_*‐only model. SHAP‐based feature‐importance analysis was used to interpret the contribution of each mechanical marker to the model prediction (Figure 6B). Feature‐importance analysis in atherosclerotic lesions revealed that *D_creep_*, *τ_1/2_*, *E_sum_*, and *η* were the strongest predictors at different stages.

A larger mean absolute SHAP value indicates that the corresponding mechanical marker contributes more strongly to the model's classification decision. This analysis was used to determine whether classification relied primarily on *E_exp_* alone or on a broader set of complementary mechanical markers such as creep, relaxation, and viscoelastic model‐derived parameters. Although *E_exp_* alone allowed partial discrimination of plaques across early‐, mid‐, and late‐stage (Figure 6C–E), substantial overlap persisted between the 12‐ and 20‐week groups (Figure 6F,I,L). Region‐specific classification further demonstrated that LP could be identified with near‐perfect accuracy using both single‐modulus and multi‐parameter models (Figure 6L–N). CF and IF also displayed strong separability due to their consistently high stiffness and slow relaxation behavior (Figure 6F–K). Collectively, these results demonstrate that multiscale viscoelastic parameters encode robust, classifiable mechanical fingerprints that can be effectively decoded by the EnsNN.

To determine whether multiscale mechanical parameters could distinguish the structurally heterogeneous regions emerging during middle‐stage atherosclerosis (12‐week sample), we applied the EnsNN classifiers to CF, IF, and LP datasets in 12‐week groups. Feature‐importance analysis identified *E_exp_*, *α_long_*, and *η* as the dominant predictors for the overall three‐class model (Figure 6B), underscoring the central role of ECM stiffness and long‐timescale creep in determining regional identity. Classification performance reflected these mechanistic distinctions. *E_exp_* had high normalized importance, but when using the *E_exp_* index alone, the classification of CF, IF, and LP regions showed substantial overlap. The incorporation of all hierarchical viscoelastic parameters markedly improved classification accuracy (Figure 7), though LP remained the least separable group (Figure 7A–C). In pairwise analyses, LP vs CF or IF showed moderate classification accuracy (Figure 7G–L), whereas CF vs IF achieved near‐perfect separation (Figure 7D–F). Together, these results reveal that CF, IF, and LP in mid‐stage also possess distinct multiscale mechanical fingerprints, and that ECM stiffness and slow‐creep dynamics are key determinants of regional classification in mid‐stage.

**FIGURE 7:**
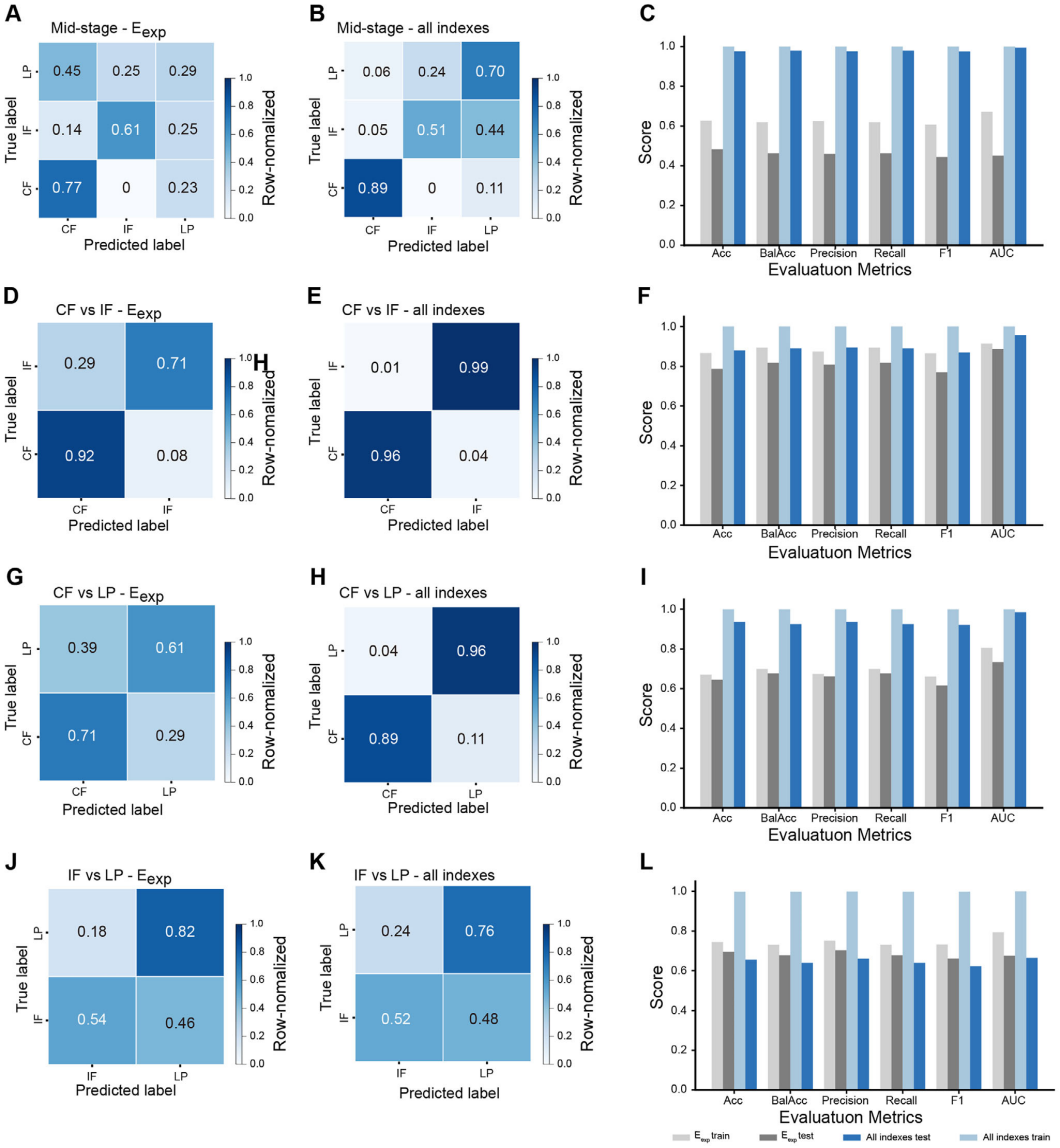
Plaque region‐specific classification of mid‐stage atherosclerotic lesions using multiscale mechanical parameters and EnsNN modeling. (A–C) Three‐class classification of mid‐stage plaque regions, including CF, IF, and LP, using *E*_exp_ alone (A) or the full multiscale mechanical‐parameter set (B), with corresponding performance metrics summarized in C. (D‐L) Pairwise regional classification within mid‐stage plaques, including CF versus IF (D‐F), CF versus LP (G‐I), and IF versus LP (J‐L). The full multiscale mechanical‐parameter set improves regional discrimination relative to *E*_exp_ alone, particularly for CF‐related comparisons, while IF‐LP separation remains comparatively limited, suggesting partial mechanical overlap between these two plaque regions at the mid stage.

To determine whether late‐stage (20‐week sample) plaques exhibit enhanced mechanical divergence across regions, we also applied EnsNN classifiers on CF, IF, and LP regions at 20 weeks. Feature‐importance analysis showed that *τ_1/2_*, *η*, and *E_exp_* were the most influential predictors (Figure 6B), indicating that viscoelastic relaxation dynamics—not stiffness alone—dominated regional discrimination at this stage. By *E_exp_* alone, CF, IF, and LP showed extensive overlap (Figure 8A–C). Incorporating all hierarchical parameters substantially improved classification performance (Figure 8B,E,H,K). Pairwise analyses showed that CF vs IF benefited markedly from multiscale features (Figure 8D–F), whereas CF vs LP achieved near‐perfect separability (Figure 8G–I), reflecting their opposite mechanical phenotypes. IF vs LP classification improved with full‐parameter models but remained partially overlapping (Figure 8J–L), highlighting the mechanical similarity of these soft, highly remodeled regions.

**FIGURE 8:**
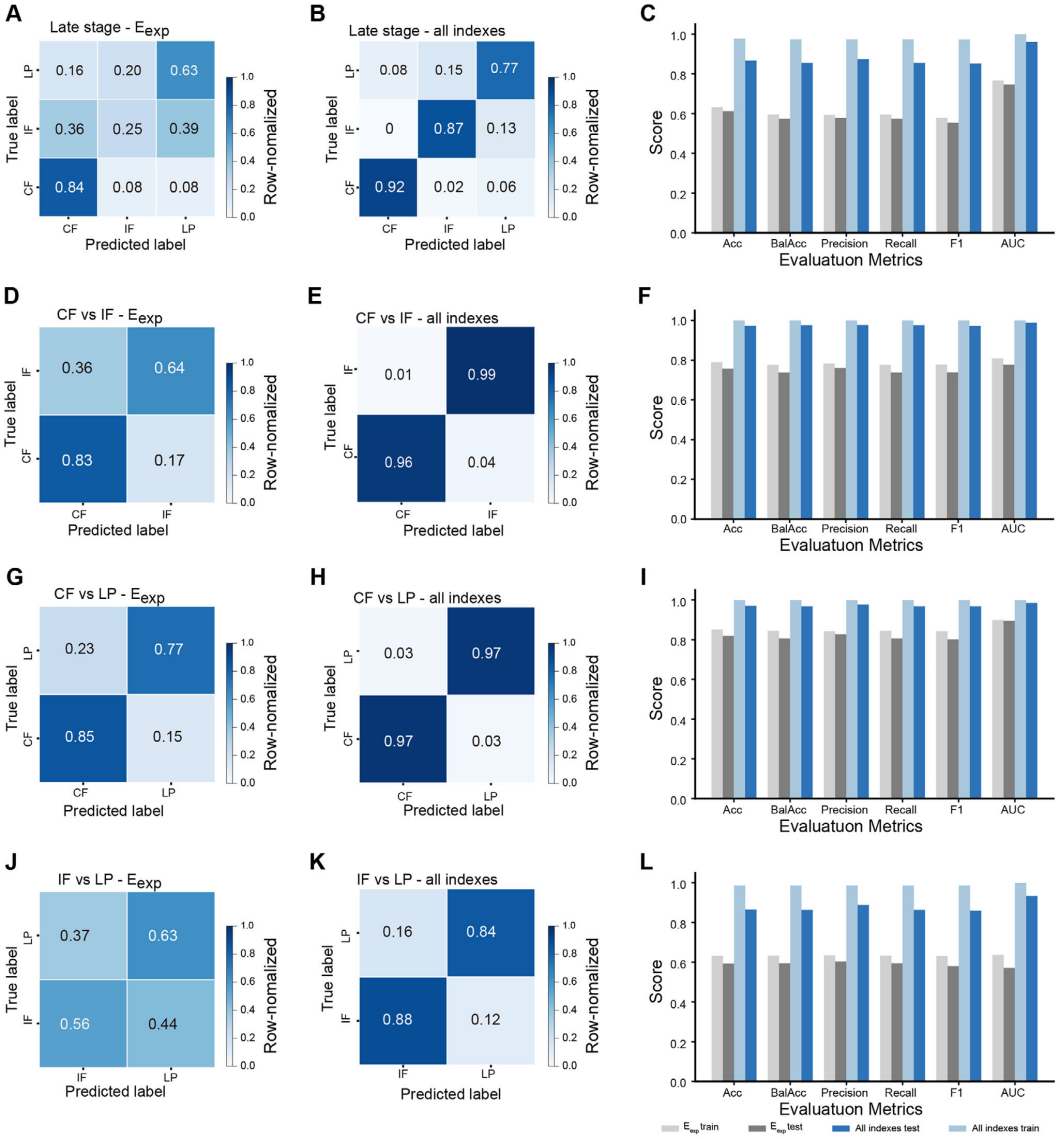
Plaque region‐specific classification of late‐stage atherosclerotic lesions using multiscale mechanical parameters and EnsNN modeling. Regional classification was performed within 20‐week plaques to evaluate whether multiscale mechanical signatures could resolve late‐stage plaque heterogeneity. (A–C) Three‐region classification among CF, IF, and LP using *E*_exp_ alone (A) or the full multiscale mechanical‐parameter set (B), with corresponding evaluation metrics shown in C. (D–L) Pairwise regional classification for CF versus IF (D–F), CF versus LP (G–I), and IF versus LP (J–L). Compared with the *E*_exp_ alone model, the all‐index EnsNN model substantially improved regional separation across late‐stage plaque components, indicating that the combined viscoelastic parameter set captures region‐specific mechanical fingerprints that are not fully represented by apparent elastic modulus alone.

These parameters integrate collagen density, fiber alignment, crosslinking, and transverse expansion stiffness, thereby reflecting the macroscopic load‐bearing capacity of each region. The excellent separability of CF and LP aligns with their distinct structural states: CF exhibits densified collagen networks and slow relaxation, while LP features lipid‐rich, weakly constrained, rapidly relaxing environments. In contrast, IF displays mixed mechanical characteristics, consistent with its heterogeneous cellularity and partially degraded ECM, explaining its intermediate and more error‐prone classification.

Machine learning effectively integrates these axes to identify biomechanical “fingerprints” associated with distinct plaque phenotypes. Such signatures may serve as quantitative markers for risk stratification and could inform future development of imaging‐based mechanical diagnostics. The predictive power of the EnsNN underscores that hierarchical mechanical parameters provide the most informative representation of atherosclerotic lesions.

## 3 Conclusion

In this study, we established a multiscale viscoelastic characterization framework to investigate the spatiotemporal mechanical remodeling of atherosclerotic plaques during disease progression. By integrating static elasticity, creep, stress relaxation, hierarchical viscoelastic modeling, and two‐stage power‐law rheology, we demonstrated that plaque mechanics evolve in a stage‐ and region‐dependent manner rather than through uniform stiffening. Dynamic viscoelastic parameters, including *D_creep_*, *τ_1/2_*, *α_short_*, and *α_long_*, together with hierarchical mechanical parameters derived from the HHM model, provided complementary information beyond the conventional elastic modulus and revealed progressive mechanical heterogeneity associated with plaque maturation [18, 20]. Previous AFM studies have similarly demonstrated marked mechanical heterogeneity among fibrous, lipid‐rich, and other plaque components, supporting the use of localized mechanical signatures to characterize plaque composition [18, 20, 45].

The multiscale mechanical signatures were further integrated with histological and inflammatory analyses, demonstrating that mechanical remodeling closely accompanies changes in plaque composition and inflammatory status throughout disease progression. These findings suggest that combining elastic and time‐dependent viscoelastic descriptors provides a more comprehensive biomechanical characterization of atherosclerotic lesions than stiffness measurements alone. Nevertheless, the associations between inflammatory remodeling and mechanical phenotypes identified here remain correlative and require further mechanistic validation.

To evaluate the potential utility of these mechanical descriptors, we implemented an ensemble neural‐network classifier integrating elastic, viscoelastic, and rheological parameters. Compared with individual mechanical indices, the multivariate model showed improved discrimination of plaque stages and regions, suggesting that different plaque components possess distinct multiscale mechanical fingerprints. However, because the current study was based on ex vivo murine tissues and the available biological cohort did not permit rigorous animal‐level external validation, these machine‐learning findings should be considered exploratory rather than evidence of established diagnostic generalizability.

Several limitations should be acknowledged. First, the relationships between inflammatory remodeling and mechanical changes observed in the present study are associative and require direct causal validation. Second, AFM measurements were performed ex vivo and at the microscale, whereas clinical imaging operates at substantially larger spatial scales and under physiological loading conditions. Therefore, direct equivalence between AFM‐derived parameters and in vivo elastographic measurements cannot currently be assumed. Third, larger animal cohorts, human specimens, grouped validation strategies, and cross‐modality comparisons will be required to establish the generalizability and translational relevance of the proposed mechanical framework.

Although AFM itself is currently restricted primarily to ex vivo microscale mechanical characterization, the comprehensive stage‐ and region‐resolved mechanical information obtained here may provide a useful reference for future cross‐modality studies. Ultrasound‐based vascular elastography has been applied to characterize the mechanical properties of carotid arteries and atherosclerotic plaques, including plaque strain, shear‐wave velocity, and stiffness‐related parameters [46, 47, 48, 49, 50, 51, 52, 53, 54, 55]. In particular, shear‐wave elastography has demonstrated the ability to differentiate plaque mechanical phenotypes and has shown associations between shear‐wave measurements and plaque composition or vulnerability‐related features [53, 54, 55]. Marlevi et al. further demonstrated that combined spatiotemporal and frequency‐dependent shear‐wave elastography could differentiate vulnerable from stable carotid plaques, with shear‐wave metrics associated with lipid‐rich necrotic core content, fibrous‐cap characteristics, and other plaque components [55].

Optical coherence elastography (OCE) provides another potential bridge between microscale biomechanical measurements and clinically accessible intravascular imaging. OCE extends OCT by mapping mechanically induced tissue deformation and can therefore provide information related to local biomechanical properties [56]. Intravascular OCE has already been demonstrated using catheter‐based systems capable of resolving circumferential mechanical contrast in arterial tissues [43]. Accordingly, rather than directly translating AFM measurements into clinical diagnostic parameters, a more realistic future strategy would be to establish quantitative relationships between microscale AFM‐derived mechanics and macroscale or mesoscopic measurements obtained using ultrasound elastography, shear‐wave elastography, OCT/OCE, or other biomechanical imaging modalities. Establishing such cross‐scale calibration standards may enable clinically accessible imaging techniques to indirectly reflect microscale plaque mechanical states identified by AFM.

Overall, this work establishes a multiscale viscoelastic framework for characterizing the spatiotemporal evolution of atherosclerotic plaque mechanics. By providing detailed ex vivo mechanical benchmarks across disease stages and plaque regions, the present study may serve as a foundation for future efforts to link microscale AFM measurements with clinically accessible biomechanical imaging and to develop mechanically informed approaches for atherosclerosis assessment.

## 4 Materials and Methods

### 4.1 Animal Culture and Sample Preparation

Apolipoprotein‐E‐deficient (ApoE^−/−^) mice on a C57BL/6 background and C57BL/6 wild‐type (WT) controls (Charles River Laboratories, Wilmington, MA, USA) were used. All procedures conformed to institutional guidelines and were approved by the Northwestern Polytechnical University Institutional Animal Care and Use Committee (protocol approved; details available upon request). Animals were maintained in a specific‐pathogen‐free facility on a 12‐h light/dark cycle (temperature 22 ± 2°C; relative humidity 40–60%) with food and water ad libitum.

To capture stage‐wise plaque development, tissues were collected at week 0 (baseline) and weeks 2, 6, 12, and 20 relative to disease induction. Unless indicated, each experimental group included biological replicates (n ≥ 3). Mice were anesthetized according to IACUC‐approved procedures and euthanized; the arterial tree was exposed and rinsed/perfused with phosphate‐buffered saline (PBS, pH 7.4). For histology (H&E, Masson's trichrome, Von Kossa, Oil Red O), segments were fixed and processed as described in Section 4.2. For mechanical assays, adjacent serial sections were prepared on a cryostat at a nominal thickness ∼15 µm; sections designated for AFM‐based microrheology were kept hydrated during testing, and OCT residues were removed by gentle PBS rinses immediately before measurements to avoid artifacts.

### 4.2 Biological Staining

#### 4.2.1 Tissue Handling

Arterial segments were harvested as in 4.1. For paraffin histology (H&E, Masson, von Kossa), tissues were fixed in 10% neutral‐buffered formalin (≈4% formaldehyde) at room temperature (24–48 h), processed, embedded, and sectioned at 5–7 µm. For lipid‐preserving Oil Red, specimens were fresh‐frozen in OCT and cryosectioned at 6–10 µm; alcohol dehydration was avoided.

#### 4.2.2 Oil Red O

Sections were equilibrated in 60% isopropanol (30–60 s), stained in freshly filtered ORO working solution (8–12 min), briefly differentiated in 60% isopropanol (5–15 s), counterstained with hematoxylin, and mounted in aqueous medium. For en face staining of opened arteries, tissues were PFA‐fixed (4%, 15–30 min) and stained for 10–15 min. Steps followed standard protocols with minor timing optimizations.

#### 4.2.3 Hematoxylin & Eosin

Deparaffinization in xylene, graded rehydration, hematoxylin (2–5 min) and bluing, eosin (30–90 s), rapid dehydration/clearing, and resin mount were performed per vendor instructions.

#### 4.2.4 Masson

Optional Bouin's pre‐mordant, Weigert's iron hematoxylin, Biebrich scarlet–acid fuchsin, phosphomolybdic/phosphotungstic differentiation, aniline blue, 1% acetic rinse, dehydration/clearing, and mounting followed the standard method.

#### 4.2.5 von Kossa

Sections were incubated in 1% AgNO_3_ under bright light (10–30 min), fixed with sodium thiosulfate (2–5 min), counterstained, dehydrated, and mounted as in established procedures.

#### 4.2.6 Immunofluorescence

FFPE sections underwent heat‐mediated antigen retrieval (citrate pH 6.0 or Tris‐EDTA pH 9.0, 95–98°C, 15–20 min). Cryosections were briefly PFA‐fixed when needed. Permeabilization (0‐0.1% Triton X‐100) and blocking (5%–10% normal serum + 1% BSA, 1 h) were adjusted to antigen localization. Primary antibodies (CD16(Invitrogen, MA1‐7633); CD206(Abcam, ab64693); CD68(Abcam, ab53444)) were incubated overnight at 4°C; fluorophore‐conjugated secondary antibodies were applied for 1 h at room temperature; DAPI counterstain and aqueous anti‐fade mounting completed the procedure.

#### 4.2.7 Quality Control and Quantification

Acquisition settings were held constant within a batch; negative controls (isotype or secondary‐only) were included. Lipid‐positive area and macrophage markers were quantified in ImageJ/FIJI using fixed thresholds; technical replicates were averaged per site, then per animal, before statistics to avoid pseudo‐replication.

### 4.3 Static Mechanical and Dynamical Characterizations

In this study, tissue samples from 3 animals within each experimental group were pooled for AFM characterization. As a result, individual measurements could not be assigned to specific animals, and animal‐level data partitioning was therefore not feasible, but Static mechanical and Dynamical characterizations were measured in the same spot more than twice.

#### 4.3.1 Single Force–Distance Curve Maps

We quantified quasi‐static tissue stiffness from AFM micro‐indentation on regions co‐registered with histology. A custom‐modified spherical probe was used for all experiments, consisting of an NP‐O10 tipless cantilever (Bruker, Camarillo, CA, USA) attached to a 20 µm diameter silicon dioxide sphere (Fuhong Nanomaterials, China). Before AFM measurements, frozen samples were thawed for 15 min at room temperature and washed with ultra‐pure water for 10 min to remove any OCT compound. The tissues were then immersed in a liquid chamber during the experiments.

Before performing mechanical tests, the spring constant (k) and resonance frequency (f) of the probe were calibrated (k = 0.35 N/m, f = 18 kHz). Prior to measurements, the deflection inverse optical lever sensitivity (DeflInvOLS) was calibrated from the slope of the deflection‐Z displacement curve to convert photodiode voltage signals into cantilever deflection, and DeflInvOLS remained stable at approximately 100 nm/V throughout the experiments. The calibrated spring constant and Defl InvOLS values were subsequently used for quantitative force measurements. To ensure a comprehensive evaluation of tissue mechanical changes and minimize the influence of local heterogeneity, measurements were taken at multiple locations, each separated by more than 200 µm within each region. Additionally, a relatively large spherical probe (diameter = 20 µm) was used to assess localized tissue mechanical properties at the micron scale, instead of focusing on individual tissue components like extracellular fibers or cells with a nanoscale AFM probe. The initial contact point during the experiment was triggered when the cantilever deflection voltage reached 1 V, which corresponded to an initial cantilever deflection of approximately 100 nm and the applied force of approximately 35 nN. For each location, load‐displacement curves (approached at *v* = 1 µm/s and gradually pressed into the sample until reaching the predetermined maximum force of 20 nN) were fitted using a spherical‐contact Hertz model with finite‐thickness correction to obtain the apparent Young's modulus *E_exp_*. The resulting per‐sample distribution of *E_exp_* was summarized by robust statistics (median) and propagated to group‐level comparisons. Actually, the indentation depth is 602.9 ± 624.2 nm (mean ± SD) in the elasticity modulus experiment.

Quality control. AFM measurements were performed at multiple sites per region. Randomization was implemented as follows: measurement locations were selected using randomized regions but assisted by biological staining; during data processing, analysts were blinded to group assignment (stages/regions). The total number of sites for each comparison is reported in Table S6.

Each map contained (≥30) sites, covering (≥15) per region. Site‐level outliers (median absolute deviation > 3.5) were removed before computing per‐region summaries.

The deflection of the cantilever (*σ*_deflection_) was measured by tracking the vertical position change of a laser beam reflected off the cantilever's backside. The Z‐sensor displacement corresponds to the overall vertical travel distance of the AFM probe (Z) during indentation and was monitored by the AFM system. The probe indentation depth (*h*) was then calculated as:

(4) $$h=Z-\sigma_{deflection}$$

The applied force on the sample (F) was determined using Hooke's law:

(5) $$\begin{array}{c} F=k\cdot \sigma_{\mathrm{deflection}} \end{array}$$

The sample deformation corresponding to the probe indentation depth (*h*) was calculated as:

(6) $$\begin{array}{c} h=Z-\frac{F}{k} \end{array}$$

where k is the spring constant of the cantilever. For all measurements, the IGOR Pro software (Version 17, Asylum Research, Oxford Instruments, USA) was used to determine the elastic modulus (*E*) using the Hertz model:

(7) $$\begin{array}{c} E=\frac{3F\left(1-v^{2}\right)}{4\sqrt{R}h^{\frac{3}{2}}}\; \end{array}$$

where v is Poisson's ratio (taken as 0.5 for incompressible materials), and R is the radius of the spherical probe.

#### 4.3.2 Dynamic Mechanical Curve Maps

AFM directly records force and displacement signals rather than spatially resolved stress and strain fields. The conventional relation *J* (*t*) =  ε(*t*)/σ(*t*) represents the classical bulk‐rheology definition of creep compliance, but it was not used to calculate local stress or strain in the present indentation experiments. Because the stress and strain fields beneath a spherical indenter are spatially nonuniform, the dynamic mechanical quantities were instead derived from.

##### 4.3.2.1 Constant‐Force Creep

The triggering conditions for the experiment are consistent with the static mechanical measurements. During the creep protocol, force feedback maintained the applied force at approximately *F*_0_ =  20 nN for 10 s, while the time‐dependent indentation response *h*(*t*)was recorded. For a rigid spherical indenter and a linear viscoelastic material with monotonically increasing contact area, the Hertz contact relation combined with the Boltzmann superposition principle gives.

(8) $$F\;\left(t\right)=\frac{4\sqrt{R}}{3\left(1-\nu^{2}\right)}\;\int_{0}^{t}E\left(t-\tau \right)\frac{d}{d\tau }\left[h{\left(\tau \right)}^{3/2}\right]d\tau$$

where *R*is the probe radius, νis Poisson's ratio, and *E*(*t*)is the effective relaxation modulus. For an approximately step‐like constant force, *F* (*t*) = *F*_0_ *H*(*t*), the corresponding indentation‐derived effective creep compliance is

(9) $$J_{\mathrm{ind}}\;\left(t\right)=\frac{4\sqrt{R}}{3\left(1-\nu^{2}\right)F_{0}}\;h{\left(t\right)}^{3/2}$$

Thus, *J*_ind_(*t*)was derived from the experimentally measured force and indentation histories rather than from separately calculated local stress and strain fields. The resulting compliance was plotted in log–log coordinates. The short‐ and long‐timescale power‐law exponents were obtained from linear regressions over 0.01–0.1 s and 1–10 s, respectively:

(10) $$\alpha =\frac{d\log J_{\mathrm{ind}}\left(t\right)}{d\log t}\;$$

Actually, the indentation depth is 253.9 ± 312.7 nm (mean ± SD) in creep.

##### 4.3.2.2 Fixed‐Z Force Relaxation

In the relaxation protocol, the triggering conditions for the experiment are consistent with the static mechanical measurements, and the Z‐sensor was displaced by a nominal value of 1 µm and then held at a fixed position for 10 s, while the force decay *F*(*t*)was recorded. Because cantilever deflection changes during the holding phase, a fixed Z‐sensor position does not necessarily correspond to strictly constant indentation. We therefore describe this measurement as fixed‐Z force relaxation and report a curve‐derived force‐relaxation half‐time rather than a bulk relaxation modulus.

The initial force *F*_0_was defined at the beginning of the holding phase, *t*  = *t*_0_ . The terminal force *F*_end_was calculated as the mean force over the terminal stable portion of the 10‐s recording. The half‐relaxation force was defined as

(11) $$F_{1/2}=F_{\mathrm{end}}+\frac{1}{2}\;\left(F_{0}-F_{\mathrm{end}}\right)=\frac{F_{0}+F_{\mathrm{end}}}{2}\;$$

The force‐relaxation half‐time was then calculated as

(12) $$\tau_{1/2}=t\;\left[F\;\left(t\right)=F_{1/2}\;\right]-t_{0}$$

Equivalently, the normalized force‐relaxation curve was defined as

(13) $$\tilde{F}\;\left(t\right)=\frac{F\left(t\right)-F_{\mathrm{end}}}{F_{0}-F_{\mathrm{end}}}\;$$

and τ_1/2_corresponded to the time at which

(14) $$\tilde{F}\;\left(t_{0}+\tau_{1/2}\right)=0.5$$

Linear interpolation between adjacent acquisition points was used when *F*_1/2_occurred between two sampled values.

Actually, the indentation depth is 117.5 ± 7.6 nm (mean ± SD), and the obtained initial force was 132.1 ± 157.8 nN (mean ± SD) in relaxation.

The testing procedure involved two steps: static and dynamic assessments. First, the spherical probe was guided to a random position and swiftly applied to the sample at a speed of 20 µm/s, maintaining contact with a pre‐defined force of 20 nN (creep deformation) or pre‐defined Z‐sensor displacement of 1 µm (relaxation deformation) for 10 s to record the dynamic mechanical response of the sample. Subsequently, the probe was retracted at a speed of 20 µm/s to prevent tissue adhesion. Afterward, a 30‐s waiting period was introduced to allow sample stabilization before conducting static micro‐indentation at the same location. The indentation depth mostly remained below 1 µm (less than 10% of the sample thickness) to avoid substrate effects.

### 4.4 Machine Learning for Diagnosis

#### 4.4.1 Evaluation Metrics and Model Interpretation

To evaluate whether multiscale viscoelastic mechanical parameters improve the discrimination of different stages and regions of atherosclerotic plaques, machine‐learning models were constructed using AFM‐derived mechanical features. The extracted features included the elastic modulus (*E_exp_*), viscoelastic parameters (*D_creep_* and τ_1/2_), creep‐related parameters (*E*_1_ *E*_1_, *E*_2_, *E*_3_, *E_sum_*, and η), and power‐law exponents (α_*short*_ and α_*long*_).

Mechanical parameters were extracted independently from each AFM measurement site after quality control. Because different mechanical parameters were obtained using different fitting procedures, parameter‐specific quality control was performed before downstream analysis. Site‐level outliers were identified independently for each parameter using the median absolute deviation (MAD) criterion. Measurements were excluded if they showed unstable force curves, failed mechanical model fitting, poor fitting quality, missing parameter values, or were identified as MAD‐based outliers. Consequently, the number of retained measurements differed among mechanical parameters. The numbers of initial measurements and retained measurements for each parameter are summarized in Table S6.

The machine‐learning analysis was performed using individual AFM measurement sites after parameter‐specific quality control.

Nine classification models were evaluated, including Decision Tree, Gaussian Naïve Bayes, Logistic Regression, Linear Support Vector Machine (Linear SVM), Radial Basis Function Support Vector Machine (RBF SVM), Random Forest, k‐Nearest Neighbor (KNN), Single Neural Network, and the proposed Ensemble Neural Network (EnsNN). All models were implemented using identical feature sets and preprocessing procedures to ensure a fair comparison.

Model performance was evaluated using repeated stratified five‐fold cross‐validation. In each repetition, the dataset was randomly divided into five stratified folds while preserving the class distribution. Four folds were used for model training, and one fold was reserved for testing. This procedure was repeated ten times using different random seeds, yielding fifty independent held‐out evaluations for each classification task. The same cross‐validation partitions were applied to all models.

For each classification task, predictions were summarized using the confusion matrix. Overall accuracy was calculated as accuracy. Because the number of measurements differed among classes, balanced accuracy and macro‐F1 were selected as the primary evaluation metrics.

Micro‐averaged metrics and weighted F1‐score were also calculated as supplementary performance indicators but were not used for model selection because, for single‐label classification, micro‐averaged metrics are mathematically equivalent to overall accuracy, whereas weighted metrics are dominated by majority classes.

The discriminative ability of each model was further evaluated using the area under the receiver operating characteristic curve (AUC). For multiclass classification, the macro‐averaged one‐versus‐rest (macro‐OVR) strategy was adopted,

(15) $$AU\;C_{macro-OVR}=\frac{1}{K}\;\sum_{k=1}^{K}AUC_{k}^{one-vs-rest}$$

To evaluate model generalization, the train‐test performance gap was calculated as

(16) $$Gap=Score_{train}-Score_{test}$$

and the macro‐F1 gap was used as the primary indicator of potential overfitting,

(17) $$Ga\;p_{F1}=F1_{macro,train}-F1_{macro,test}$$

The stability of model performance was assessed using the standard deviation of the test scores obtained from repeated cross‐validation,

(18) $$StabilitySD=SD\left(S,c,ore_{test}^{\left(1\right)}\text{…}Score_{test}^{\left(R\right)}\right)$$

where *R*  =  50.

Finally, SHAP (SHapley Additive exPlanations) analysis was performed to quantify the contribution of each mechanical feature to the classification results [57]. Feature importance was ranked according to the mean absolute SHAP value, allowing interpretation of whether classification relied primarily on elastic modulus or on complementary viscoelastic, creep, and relaxation parameters.

In this study, the mean absolute SHAP value was used to rank feature importance. A larger mean absolute SHAP value indicates that the corresponding mechanical marker contributes more strongly to the model's classification decision. This analysis was used to determine whether classification relied primarily on *E*_exp_alone or on a broader set of complementary mechanical markers such as creep, relaxation, and viscoelastic model‐derived parameters.

#### 4.4.2 Machine Learning

Mechanical marker data were collected from nine biological experimental groups: 0 week, 2 weeks, 6 weeks, 12 weeks‐CF, 12 weeks‐IF, 12 weeks‐LP, 20 weeks‐CF, 20 weeks‐IF, and 20 weeks‐LP. Based on these groups, we constructed a series of binary and multi‐class classification tasks to evaluate whether the measured mechanical markers could distinguish different disease stages and plaque locations. These tasks included stage classification, pairwise comparisons between 12‐week and 20‐week samples at the same plaque location, and plaque‐location classification within the 12‐week or 20‐week groups. For the main feature‐level comparison, two feature configurations were considered. The first used only the apparent elastic modulus *E*_exp_, which served as a representative single mechanical marker. The second used the full mechanical‐marker panel, including *E_exp_*, *D*_creep_, α_short_, α_long_, *E*_1_, *E*_2_, *E*_3_, *E*_sum_, η, and *τ_1/2_*. This comparison was designed to determine whether the combined viscoelastic marker panel provided additional diagnostic information beyond *E*_exp_ alone.

All preprocessing steps were performed within each training fold to avoid preprocessing‐related information leakage. Missing values were imputed using the median value estimated from the training fold only. Mechanical‐scale variables were log‐transformed when appropriate and then standardized using the mean and standard deviation of the training fold. The same transformations were subsequently applied to the corresponding held‐out test fold. The primary classifier was an ensemble softmax feed‐forward neural network. Each subnetwork was a shallow neural network consisting of an input layer, one hidden layer with 8 ReLU units, and a task‐specific softmax output layer. The number of output neurons was equal to the number of classes in the corresponding task. The network was trained using class‐weighted cross‐entropy loss with *L*_2_weight regularization:

(19) $$L=-\sum_{i=1}^{n}w_{y_{i}}\log p\left(y_{i}|x_{i}\right)+\lambda ∥\theta {∥}_{2}^{2}$$

where $w_{y_{i}}$ is the class weight, *p*(*y_i_* ∣*x_i_*)is the predicted probability for the true class, θdenotes the trainable parameters, and λis the regularization coefficient. To reduce sensitivity to random initialization, the final model was constructed as an ensemble of *N*  =  12independently initialized subnetworks. The final class probability was obtained by averaging the softmax outputs of all subnetworks:

(20) $$p_{\mathrm{ens}}\left(y=k|x\right)=\frac{1}{N}\;\sum_{m=1}^{N}p_{m}\left(y=k|x\right)$$

and the predicted class was assigned as the class with the highest averaged probability.

To determine whether the neural‐network model was justified relative to simpler classifiers, we compared it with several baseline models, including logistic regression, linear support vector machine, radial‐basis‐function support vector machine, k‐nearest neighbors, decision tree, random forest, and Gaussian naive Bayes. A single softmax neural network was also evaluated to assess the benefit of ensembling.

All models were trained and tested using the same feature sets, preprocessing steps, and cross‐validation splits. Model performance was evaluated using repeated stratified five‐fold cross‐validation. In each repetition, the dataset was divided into five stratified folds. Four folds were used for training, and the remaining fold was used as the held‐out test set. This process was repeated 10 times with different random seeds, resulting in 50 held‐out test evaluations for each model and classification task. For every fold, both training and test performance were recorded, allowing the train‐test performance gap to be quantified. The primary evaluation metrics were accuracy, balanced accuracy, macro‐precision, macro‐recall, macro‐F1, and macro‐averaged one‐vs‐rest AUC. Macro‐F1 and balanced accuracy were emphasized because they assign equal importance to each class and are less affected by class imbalance. Micro‐averaged metrics were not used as primary criteria because, for single‐label classification, micro‐precision, micro‐recall, and micro‐F1 are mathematically equivalent to overall accuracy. Weighted metrics were reported as supplementary information but were not used as the main criterion because they are dominated by majority classes and can mask poor performance in minority classes.

To further evaluate the effect of ensemble size, we repeated the analysis using *N*  =  1, 2, 3, 5, 8, 10, 11, 12 *and* 15subnetworks. Model selection was based on the overall trade‐off among held‐out test performance, macro‐F1, balanced accuracy, train‐test generalization gap, and repeat‐to‐repeat stability. Confusion matrices were generated using only held‐out test predictions from the repeated cross‐validation procedure. Feature importance was further assessed using SHAP‐based analysis to identify mechanical markers that contributed most strongly to classification.

### 4.5 Statistical Analysis

All data were presented as mean ± standard deviations (SDs) and displayed using violin, box and whisker plots, where the whiskers extended to the most extreme data points within 1.5 × the interquartile range (IQR). The normality of data was assessed using the Shapiro‐Wilk test. Comparisons among different plaque components were performed using the Kruskal–Wallis nonparametric test, followed by multiple‐comparison correction using the two‐stage step‐up method of Benjamini, Krieger, and Yekutieli. The distributions of all parameters between different regions were compared using the two‐sample Kolmogorov‐Smirnov (K‐S) test. Specifically, the two‐sample K‐S test involves formulating hypotheses to compare the distributions of two samples, calculating the empirical distribution functions (EDFs) for each sample, and computing the test statistic D as the maximum difference between the EDFs. The p‐value is then determined based on D and the sample sizes. Finally, the p‐value is compared to a significance level to decide whether to reject the null hypothesis that the samples come from the same distribution. This statistical analysis enabled us to comprehensively characterize and compare the mechanical properties across various plaque regions, providing valuable insights into the mechanical heterogeneity within atherosclerotic plaques. All histogram data were analyzed using a Lorentzian fit to assess the distribution profiles and peaks of the mechanical indices for different plaque regions. To evaluate the discriminative performance of the proposed indices in distinguishing various atherosclerotic regions, nonparametric receiver operating characteristic (ROC) curves were employed. The effectiveness of each index was quantitatively assessed by calculating the area under the ROC curve (AUC). Statistical analyses and ROC evaluations were performed using GraphPad Prism version 9 (GraphPad Software, San Diego, California USA) and OriginPro (OriginPro, Version 2024, OriginLab Corporation, Northampton, MA, USA). A significance level of *p* < 0.05 was considered statistically significant.

## Author Contributions

Conceptualization: Y.D.Z., N.Z., and H.Y.; Methodology: Y.D.Z., Z.C., X.J.W., H.Y.X., Z.W., and G.K.X.; Investigation: Y.D.Z., S.J.W., Y.Z.Z., and R.N.P.; Visualization: Y.D.Z., H.Z., H.Y.X., N.Z., and H.Y.; Supervision: H.Y.X., Y.L., G.K.X., N.Z., and H.Y.; Writing – original draft: Y.D.Z. and N.Z.; Writing – review & editing: Y.D.Z., Z.C., Y.L., H.Y.X., G.K.X., N.Z., and H.Y.; Funding acquisition: H.Y., N.Z., Y.L., and Y.Z.Z.

## Funding

This work was supported by the National Natural Science Foundation of China (Grant Nos. 12002285), the National Science Foundation of Shaanxi (Grant Nos. 2025JC‐YBMS‐044), the funding for clinical trials from the affiliated Drum Tower Hospital, Medical School of Nanjing University (Grant Nos. 2024‐LCYJ‐MS‐10), and the Innovation Foundation for Doctor Dissertation of Northwestern Polytechnical University (Grant Nos. CX2025102).

## Code Availability

The source code has been deposited in GitHub (https://github.com/yidanzhou821‐collab/ML_FOR_MECHANIC_INDEX).

## Conflicts of Interest

The authors declare no conflicts of interest.

## Supporting information




**Supporting File**: advs77995‐sup‐0001‐SuppMat.docx.

## Data Availability

The data that support the findings of this study are available from the corresponding author upon reasonable request.
