# Supplementary material for: A Hybrid Hierarchical Framework for Quantifying Mechanical Markers in Atherosclerotic Disease Progression: A New Approach for Diagnosis and Risk Assessment
Source: Adv Sci (Weinh). 2026 Sep 30:e77995. Online ahead of print. doi: 10.1002/advs.77995 (PMC13626170; doi:10.1002/advs.77995)
Supplement: Supplementary file 1 [file ADVS-9999-e77995-s001.docx]

Supporting Information

Title: A Hybrid Hierarchical Framework for Quantifying Mechanical Markers in Atherosclerotic Disease Progression: A New Approach for Diagnosis and Risk Assessment

Yidan Zhou^1,2,3#^, Zhuo Chang^4#^, Xianjun Wu^4^, Sijie Wang^1,2,3^, Yuzhi Zhang^1,2,3^, Hao Zhang^1,2,3^, Haoyu Xu^5^, Ruining Peng^1,2,3^, Zhe Wang^1^, Huiyun Xu^1,2,3^, Yu Liu^6*^, Guangkui Xu^4*^, Nu Zhang^1,2,3*^, Hui Yang^1,2,3*^

**Figure S1** Representative controlled applied force and schema chart during creep measurements. (A) Representative controlled applied force during creep measurement. (B) Schema chart of time-force during creep measurements. (C) Schema chart of time-Height during creep measurements.

Figure S2 Representative controlled applied force and schema chart during relaxation measurements. (A) Representative controlled applied force during relaxation measurement. (B) Schema chart of time-force during creep measurements. (C) Schema chart of time-Height during relaxation measurements.

**Figure S3** Representative dynamic creep responses over time, fitted using our proposed theory.

**Figure S4** Self-similar hierarchical model illustrating the assessment of dynamics of plaque components from cytoplasm, cell to the tissue ECM level.

**Figure S5** Comparison of the elastic modulus of different stages and regions between experiments and a theoretical model. The comparison of the theoretically determined elastic modulus *E_sum_* and experimentally measured elastic modulus *E_exp_* for different stages and regions. No statistical differences are found.

**Figure S6** Time-dependent development of atherosclerotic lesions in *ApoE^⁻/⁻^* mice. (A) Morphology of the aorta at different stages. Representative images of whole aortas from C57 mice (top row) and *ApoE^⁻/⁻^* mice (bottom row) at 0, 2, 6, 12, and 20 weeks of high-fat diet. *ApoE^⁻/⁻^* mice show progressive lipid accumulation and plaque formation (red-stained regions). Scale bar = 1 cm. (B) Quantification of aortic area and plaque area. Black bars represent the total aortic area, while red bars represent plaque area in *ApoE^⁻/⁻^* mice. (C) Histological cross-sections of the aortic root. H&E-stained transverse sections from C57 (top row) and *ApoE^⁻/⁻^* (bottom row) mice show gradual plaque thickening, luminal narrowing, and lipid-rich core formation in *ApoE^⁻/⁻^* mice over time (n = 3 each). Scale bar = 200 µm. (D) Serum lipid profiles. Time-course measurements of Total Cholesterol (TC), Triglyceride (TG), Low-Density Lipoprotein Cholesterol (LDL), and High-Density Lipoprotein Cholesterol (HDL) in C57 and *ApoE^⁻/⁻^* mice. *ApoE^⁻/⁻^* mice exhibit significantly elevated TC, TG, and LDL levels, along with reduced HDL, consistent with hyperlipidemia-induced plaque formation (n = 5 each). (****p* < 0.001.)

**Figure S7** Temporal immunohistochemical characterization of vascular smooth muscle cells and endothelial cells during atherosclerosis progression. Representative immunohistochemical staining of vascular smooth muscle cells (VSMCs) and endothelial cells in arterial cross-sections from C57 mice and *ApoE^⁻/⁻^* mice at 0, 2, 6, 12, and 20 weeks, respectively. VSMCs are identified by positive staining for smooth muscle markers (upper rows), while endothelial cells are visualized by endothelial marker immunoreactivity (lower rows). Brown staining indicates positive expression, and nuclei are counterstained in blue. In C57 mice, the vascular structure remains largely intact with well-organized VSMC layers and continuous endothelial coverage across all time points. In contrast, *ApoE^⁻/⁻^* mice display progressive structural remodeling characterized by disorganization and depletion of VSMCs, accompanied by endothelial disruption and luminal irregularity, which become increasingly pronounced at 12 and 20 weeks (n = 3 each). Scale bars, 50 μm.

**Figure S8** Comparative performance of machine-learning models across different atherosclerotic classification tasks.

**Figure S9** Effect of ensemble size on model stability and generalization performance. Relationship between ensemble size (N) and model robustness.

**Table S1** Mean values, Coefficients of variation (CV) and centers of data distribution of elastic modulus for different stages and regions.

|  | 0 w | 2 w | 6 w | 12 w-CF | 12 w-IF | 12 w-LP | 20 w-CF | 20 w-IF | 20 w-LP |
| --- | --- | --- | --- | --- | --- | --- | --- | --- | --- |
| n | 47 | 39 | 54 | 36 | 49 | 35 | 85 | 91 | 69 |
| Median | 18.54 | 9.86 | 5.43 | 3.85 | 9.37 | 3.03 | 9.29 | 10.06 | 5.54 |
| Mean | 20.28 | 11.32 | 9.85 | 6.15 | 11.83 | 5.78 | 12.06 | 11.43 | 6.55 |
| SD | 7.9392 | 4.6336 | 9.9650 | 6.4618 | 6.9738 | 5.5057 | 9.5397 | 6.6627 | 4.5292 |
| CV | 39.14% | 40.92% | 101.18% | 105.06% | 58.93% | 95.3% | 79.13% | 58.31% | 69.12% |
| Centers of data distribution (Lorentzian fit) and R^2^ values | 17.5  (0.79) | 9.75  (0.94) | 2.04  (0.81) | 4.37  (0.98) | 9.97  （0.86） | 2.54  （0.76） | 7.39;  32.49  (0.76) | 13.79  (0.99) | 4.83  (0.99) |

**Table S2.** Mean values, Coefficients of variation (CV) and centers of data distribution of creep displacement (*D_creep_*) for different plaque regions.

|  | 0 w | 2 w | 6 w | 12 w-CF | 12 w-IF | 12 w-LP | 20 w-CF | 20 w-IF | 20 w-LP |
| --- | --- | --- | --- | --- | --- | --- | --- | --- | --- |
| n | 116 | 58 | 116 | 58 | 34 | 37 | 58 | 58 | 40 |
| Median | 80.00 | 43.45 | 103.4 | 291.5 | 297.0 | 315.0 | 390.0 | 297.6 | 660.0 |
| Mean | 92.33 | 61.67 | 152.6 | 346.8 | 371.7 | 400.3 | 416.2 | 477.0 | 717.3 |
| SD | 51.09 | 46.62 | 111.1 | 280.6 | 345.3 | 222.7 | 289.7 | 431.8 | 454.4 |
| CV | 55.33% | 75.60% | 72.82% | 80.91% | 92.90% | 55.64% | 69.59% | 90.53% | 63.35% |
| Centers of data distribution (Lorentzian fit) and R^2^ values | 154  (0.99) | 97  (0.99) | 180  (0.99) | 295  (0.99) | 103；891  （0.28） | 110；639  （0.99） | 273  (0.91) | 110;  1116  (0.89) | 112;1114(0.79) |

**Table S3.** Mean values, Coefficients of variation (CV) and centers of data distribution of relax displacement (*τ_1/2_*) for different plaque regions.

|  | 0 w | 2 w | 6 w | 12 w-CF | 12 w-IF | 12 w-LP | 20 w-CF | 20 w-IF | 20 w-LP |
| --- | --- | --- | --- | --- | --- | --- | --- | --- | --- |
| n | 46 | 34 | 29 | 22 | 36 | 29 | 40 | 25 | 24 |
| Median | 0.5820 | 0.3765 | 0.3735 | 0.4656 | 0.1190 | 0.3187 | 0.9770 | 0.7525 | 0.8128 |
| Mean | 0.6424 | 0.3839 | 0.3832 | 0.4900 | 0.2451 | 0.2847 | 0.9937 | 0.7566 | 0.8246 |
| SD | 0.3098 | 0.0757 | 0.1287 | 0.1850 | 0.1498 | 0.1141 | 0.0881 | 0.0487 | 0.0888 |
| CV | 48.22% | 19.73% | 33.59% | 37.75% | 61.13% | 40.06% | 8.863% | 6.433% | 10.77% |

**Table S4.** Mean values, Coefficients of variation (CV) and centers of data distribution of two power-law exponents for atherosclerotic lesions.

|  | 0 w | | 2 w | | 6 w | | 12 w-CF | | 12 w-IF | | 12 w-LP | | | 20 w-CF | | 20 w-IF | | 20 w-LP | |
| --- | --- | --- | --- | --- | --- | --- | --- | --- | --- | --- | --- | --- | --- | --- | --- | --- | --- | --- | --- |
| *α_short_* |  | |  | |  | |  | |  | |  | | |  | |  | |  | |
| n | 74 | | 47 | | 106 | | 34 | | 40 | | 31 | | | 33 | | 38 | | 27 | |
| Median | 0.4403 | | 0.3911 | | 0.5477 | | 0.7435 | | 0.6108 | | 0.7045 | | | 0.5135 | | 0.4715 | | 0.5170 | |
| Mean | 0.4139 | | 0.3870 | | 0.5224 | | 0.7540 | | 0.5869 | | 0.7100 | | | 0.5516 | | 0.4534 | | 0.4459 | |
| SD | 0.1151 | | 0.1496 | | 0.1241 | | 0.1079 | | 0.135 | | 0.137 | | | 0.1199 | | 0.1416 | | 0.1982 | |
| CV | 26.12% | | 38.26% | | 22.65% | | 14.51% | | 22.10% | | 19.45% | | | 23.34% | | 30.02% | | 38.33% | |
| Centers of data distribution (Lorentzian fit) and R^2^ values | 0.2994  (0.99) | | 0.3987  (0.93) | | 0.5186  (0.99) | | 0.7021  (0.73) | | 0.5882  (0.91) | | 0.7107  (0.99) | | | 0.5203  (0.89) | | 0.4587  (0.99) | | 0.2578;  0.6851  (0.94) | |
| *α_long_* |  | |  | |  | |  | |  | |  | | |  | |  | |  | |
| n | 87 | | 57 | | 104 | | 23 | | 20 | | 41 | | | 43 | | 51 | | 41 | |
| Median | 0.1432 | | 0.1281 | | 0.1450 | | 0.2264 | | 0.2272 | | 0.2131 | | | 0.1637 | | 0.1423 | | 0.1358 | |
| Mean | 0.1436 | | 0.1311 | | 0.1438 | | 0.2190 | | 0.2130 | | 0.2033 | | | 0.1693 | | 0.1468 | | 0.1432 | |
| SD | 0.0507 | | 0.0420 | | 0.0533 | | 0.0687 | | 0.0767 | | 0.0553 | | | 0.0406 | | 0.0401 | | 0.0442 | |
| CV | 35.27% | | 32.07% | | 37.06% | | 31.37% | | 36.01% | | 27.18% | | | 24.01% | | 27.3% | | 30.83% | |
| Centers of data distribution (Lorentzian fit) and R^2^ values | 0.1485  (0.92) | | 0.1287  (0.99) | | 0.1541  (0.70) | | 0.1685;  0.2749  (0.99) | | 0.1397;  0.2654  (0.98) | | 0.2117  (0.81) | | | 0.1674  (0.99) | | 0.1449  (0.99) | | 0,1469  (0.97) | |
| *E_1_* |  | |  | |  | |  | |  | |  | | |  | |  | |  | |
| n | | 48 | | 25 | | 55 | | 32 | | 35 | | 21 | 42 | | 37 | | 39 | |  |
| Median  (Pa∙s) | | 187.3 | | 173.5 | | 353.1 | | 2649 | | 490.1 | | 390.2 | 69.25 | | 95.22 | | 74.07 | |  |
| Mean  (Pa∙s) | | 287.9 | | 288.5 | | 467.6 | | 3239 | | 745.5 | | 478.3 | 150.5 | | 97.28 | | 80.95 | |  |
| SD | | 350.2 | | 369.9 | | 397.4 | | 2162 | | 448.6 | | 263.6 | 198.1 | | 105.8 | | 85.08 | |  |
| CV | | 121.6% | | 128.2% | | 84.98% | | 66.76% | | 60.18% | | 55.11% | 131.6% | | 108.7% | | 105.1% | |  |

**Table S4 (continued).**

| *η* |  |  |  |  |  |  |  |  |  |
| --- | --- | --- | --- | --- | --- | --- | --- | --- | --- |
| n | 42 | 22 | 26 | 29 | 32 | 20 | 46 | 42 | 31 |
| Median  (Pa∙s) | 39.46 | 63.44 | 90.58 | 756.7 | 390.5 | 285.0 | 37.95 | 59.99 | 58.07 |
| Mean  (Pa∙s) | 59.33 | 76.04 | 117.6 | 716.4 | 427.7 | 292.7 | 66.07 | 71.66 | 54.61 |
| SD | 60.40 | 56.53 | 70.17 | 169.4 | 306.6 | 152.8 | 71.22 | 68.86 | 48.61 |
| CV | 101.8% | 74.34% | 59.66% | 23.64% | 71.68% | 52.22% | 107.8% | 96.09% | 89.00% |
| *E_2_* |  |  |  |  |  |  |  |  |  |
| n | 49 | 36 | 65 | 25 | 15 | 20 | 46 | 45 | 40 |
| Median (Pa) | 712.6 | 418.3 | 1211 | 3212 | 1882 | 931.8 | 314.6 | 209.1 | 157.4 |
| Mean  (Pa) | 870.4 | 867.8 | 1492 | 3272 | 2338 | 1665 | 484.6 | 294.0 | 221.5 |
| SD | 1014 | 1269 | 1050 | 1409 | 1520 | 1513 | 476.2 | 328.9 | 308.7 |
| CV | 116.5% | 146.2% | 70.35% | 43.05% | 65.00% | 90.83% | 98.27% | 111.8% | 139.4% |
| *E_3_* |  |  |  |  |  |  |  |  |  |
| n | 48 | 23 | 71 | 17 | 17 | 27 | 44 | 40 | 41 |
| Median  (Pa) | 8911 | 7617 | 7948 | 899.6 | 3207 | 3207 | 1240 | 630.1 | 722.0 |
| Mean  (Pa) | 15286 | 10925 | 11019 | 3026 | 5145 | 6512 | 1745 | 1238 | 965.6 |
| SD | 14748 | 9882 | 9859 | 4210 | 4399 | 7784 | 1449 | 1490 | 932.0 |
| CV | 96.48% | 90.46% | 89.47% | 139.1% | 85.49% | 119.5% | 83.04% | 120.4% | 96.52% |

**Table S4 (continued).**

| *E_exp_* |  |  |  |  |  |  |  |  |  |
| --- | --- | --- | --- | --- | --- | --- | --- | --- | --- |
| n | 47 | 28 | 31 | 25 | 30 | 19 | 85 | 61 | 28 |
| Median  (Pa) | 18.54 | 9.86 | 8.42 | 3.21 | 9.37 | 3.03 | 9.29 | 11.26 | 3.15 |
| Mean  (Pa) | 20.28 | 11.32 | 11.02 | 3.83 | 11.83 | 5.78 | 12.06 | 12.08 | 3.94 |
| SD | 7.94 | 4.63 | 7.89 | 1.98 | 6.97 | 5.51 | 9.54 | 7.45 | 2.08 |
| CV | 39.14% | 40.92% | 71.58% | 51.65% | 58.93% | 95.30% | 79.13% | 61.62% | 52.85% |
| *E_sum_* |  |  |  |  |  |  |  |  |  |
| n | 32 | 15 | 38 | 21 | 17 | 18 | 26 | 44 | 32 |
| Median  (Pa) | 18.88 | 10.74 | 8.54 | 5.26 | 9.09 | 4.25 | 10.17 | 8.14 | 2.32 |
| Mean  (Pa) | 19.28 | 15.86 | 10.39 | 5.23 | 10.93 | 7.19 | 17.80 | 9.36 | 3.41 |
| SD | 9.31 | 8.32 | 4.80 | 2.17 | 8.18 | 5.14 | 13.67 | 6.11 | 2.30 |
| CV | 48.27% | 52.46% | 46.20% | 41.45% | 74.86% | 71.42% | 76.84% | 65.23% | 67.56% |

**Table S5.** Primers of qPCR.

|  | Forward Sequence | Reverse Sequence |
| --- | --- | --- |
| IL-6 | TACCACTTCACAAGTCGGAGGC | CTGCAAGTGCATCATCGTTGTTC |
| IL-1β | TGGACCTTCCAGGATGAGGACA | GTTCATCTCGGAGCCTGTAGTG |
| TNF-α | GGTGCCTATGTCTCAGCCTCTT | GCCATAGAACTGATGAGAGGGAG |
| IL-10 | CGGGAAGACAATAACTGCACCC | CGGTTAGCAGTATGTTGTCCAGC |
| GAPDH | CATCACTGCCACCCAGAAGACTG | ATGCCAGTGAGCTTCCCGTTCAG |

**Table S6.** The numbers of initial measurements and retained measurements for each parameter.

| measurements | | 0 w | 2 w | 6 w | 12 w-CF | 12 w-IF | 12 w-LP | 20 w-CF | 20 w-IF | 20 w-LP |
| --- | --- | --- | --- | --- | --- | --- | --- | --- | --- | --- |
| animal | | 3 | 3 | 3 | 3 | 3 | 3 | 3 | 3 | 3 |
| elastic modulus | initial | 47 | 39 | 62 | 43 | 52 | 36 | 87 | 98 | 73 |
|  | retained | 47 | 39 | 54 | 36 | 49 | 35 | 85 | 91 | 69 |
| *D_creep_* | initial | 127 | 63 | 118 | 59 | 40 | 37 | 58 | 58 | 40 |
|  | retained | 116 | 58 | 116 | 58 | 34 | 37 | 58 | 58 | 40 |
| *τ_1/2_* | initial | 48 | 34 | 31 | 22 | 36 | 29 | 40 | 25 | 24 |
|  | retained | 46 | 34 | 29 | 22 | 36 | 29 | 40 | 25 | 24 |
| *α_short_* | initial | 74 | 59 | 118 | 42 | 52 | 43 | 37 | 38 | 39 |
|  | retained | 74 | 47 | 106 | 34 | 40 | 31 | 33 | 38 | 27 |
| *α_long_* | initial | 88 | 59 | 118 | 25 | 30 | 47 | 59 | 59 | 47 |
|  | retained | 87 | 57 | 104 | 23 | 20 | 41 | 43 | 51 | 41 |
| *E_1_* | initial | 53 | 27 | 59 | 33 | 38 | 22 | 47 | 42 | 39 |
|  | retained | 48 | 25 | 55 | 32 | 35 | 21 | 42 | 37 | 39 |
| *η* | initial | 51 | 26 | 31 | 36 | 35 | 25 | 49 | 45 | 34 |
|  | retained | 42 | 22 | 26 | 29 | 32 | 20 | 46 | 42 | 31 |
| *E_2_* | initial | 53 | 42 | 72 | 31 | 19 | 25 | 48 | 47 | 43 |
|  | retained | 49 | 36 | 65 | 25 | 15 | 20 | 46 | 45 | 40 |
| *E_3_* | initial | 55 | 27 | 75 | 19 | 21 | 33 | 49 | 45 | 42 |
|  | retained | 48 | 23 | 71 | 17 | 17 | 27 | 44 | 40 | 41 |
| *E_sum_* | initial | 32 | 15 | 38 | 21 | 17 | 18 | 26 | 44 | 32 |
|  | retained | 32 | 15 | 38 | 21 | 17 | 18 | 26 | 44 | 32 |
